# An integrated atlas of human placental development delineates essential regulators of trophoblast stem cells

**DOI:** 10.1242/dev.200171

**Published:** 2022-07-06

**Authors:** Yutong Chen, Dylan Siriwardena, Christopher Penfold, Adam Pavlinek, Thorsten E. Boroviak

**Affiliations:** 1 Department of Physiology, Development and Neuroscience, University of Cambridge, Downing Site, Cambridge CB2 3EG, UK; 2 Centre for Trophoblast Research, University of Cambridge, Downing Site, Cambridge CB2 3EG, UK; 3 Wellcome Trust – Medical Research Council Stem Cell Institute, University of Cambridge, Jeffrey Cheah Biomedical Centre, Puddicombe Way, Cambridge CB2 0AW, UK; 4 King's College London, Strand, London WC2R 2LS, UK

**Keywords:** Human development, Human trophoblast stem cells, Placenta development, Self-renewal, Trophoblast

## Abstract

The trophoblast lineage safeguards fetal development by mediating embryo implantation, immune tolerance, nutritional supply and gas exchange. Human trophoblast stem cells (hTSCs) provide a platform to study lineage specification of placental tissues; however, the regulatory network controlling self-renewal remains elusive. Here, we present a single-cell atlas of human trophoblast development from zygote to mid-gestation together with single-cell profiling of hTSCs. We determine the transcriptional networks of trophoblast lineages *in vivo* and leverage probabilistic modelling to identify a role for MAPK signalling in trophoblast differentiation. Placenta- and blastoid-derived hTSCs consistently map between late trophectoderm and early cytotrophoblast, in contrast to blastoid-trophoblast, which correspond to trophectoderm. We functionally assess the requirement of the predicted cytotrophoblast network in an siRNA-screen and reveal 15 essential regulators for hTSC self-renewal, including *MAZ*, *NFE2L3*, *TFAP2C*, *NR2F2* and *CTNNB1*. Our human trophoblast atlas provides a powerful analytical resource to delineate trophoblast cell fate acquisition, to elucidate transcription factors required for hTSC self-renewal and to gauge the developmental stage of *in vitro* cultured cells.

## Abstract (precis)

**Summary:** A continuous pseudotime trajectory of human trophoblast development from zygote to mid-gestation determines the *in vivo* counterpart of *in vitro* trophoblast cultures and identifies key regulators of human trophoblast stem cell self renewal.

## INTRODUCTION

The human embryo relies on an intimate connection to the mother. This is accomplished by the trophoblast, an extra-embryonic lineage specified in the first cell fate decision of the embryo at the 16-32 cell stage (Blakeley et al., 2015; Gerri et al., 2020; Cockburn and Rossant, 2010; Niakan and Eggan, 2013; Toyooka, 2020). By the blastocyst stage, the inside of the blastocyst consists of the inner cell mass (ICM), which subsequently segregates into epiblast (EPI) and primitive endoderm (PE) to form the embryo proper and yolk sac, respectively (Cockburn and Rossant, 2010; Nichols and Smith, 2012). The outer cells constitute trophectoderm (TE), destined to mediate embryo implantation into the endometrium of the uterus and, ultimately, to give rise to the placenta.

During embryo implantation, TE diversifies into proliferative cytotrophoblast (CTB) and primary multinucleated syncytiotrophoblast (STB) (Deglincerti et al., 2016a; Hertig et al., 1956; Ruane et al., 2022). STB drives trophoblast invasion, merges fluid filled spaces into lacunae and forms a boundary between the conceptus and maternal tissues (Bischof and Irminger-Finger, 2005; Enders et al., 1997; Norwitz et al., 2001). After implantation, the proliferative CTB expands and protrudes from the primary syncytium to give rise to placental villi, the functional subunits of the placenta for the exchange of oxygen and nutrients (Burton and Jauniaux, 2017; Enders et al., 2001; Knöfler et al., 2019). The outer layer of the placental villi consists of STB, which separates fetal from maternal circulation and secretes human chorionic gonadotropin (CGB) to sustain the pregnancy (Malassiné and Cronier, 2002; Petraglia et al., 1996). CTB cells in the periphery of the placental villi proliferate laterally and form the cytotrophoblastic shell that surrounds the conceptus, or differentiate into invasive extravillous trophoblast (EVT) cells (Knöfler et al., 2019). To prevent rejection of the conceptus by the maternal immune system, trophoblast cells exhibit a unique human leukocyte antigen (HLA) profile. CTB and STB lack HLA-A and HLA-B, whereas EVT is hallmarked by trophoblast-specific expression of HLA-G (Apps et al., 2009; Hiby et al., 1999; King et al., 2000). Despite an extensive morphological understanding of human placentation, the molecular mechanisms of early trophoblast lineage specification remain poorly understood.

Single-cell studies of human blastocysts and placentas from first and second trimester abortions have provided important insights into pre- and postimplantation placental development (Blakeley et al., 2015; Liu et al., 2018; Petropoulos et al., 2016; Vento-Tormo et al., 2018; West et al., 2019; Yan et al., 2013). In addition, the recent establishment of *in vitro* human embryo culture to early postimplantation stages has further elucidated peri-implantation trophoblast development (Deglincerti et al., 2016b; Ruane et al., 2022; Shahbazi et al., 2016; Xiang et al., 2020; Zhou et al., 2019). Although the gene regulatory network analysis performed within individual studies has revealed state-specific regulators of trophoblast differentiation (Petropoulos et al., 2016; Xiang et al., 2020; Zhou et al., 2019), there is no unified resource to study the developmental progression from trophoblast specification to placenta formation.

The derivation of human trophoblast stem cells (hTSCs) (Okae et al., 2018) from both blastocyst and first trimester placental tissue has provided a system to functionally interrogate human-specific regulatory networks. Conventional transcriptome analysis of hTSC bulk cultures suggests that hTSCs resemble first trimester CTB (Castel et al., 2020; Okae et al., 2018), but developmental staging at single-cell resolution has remained elusive. The most recent advances use naïve human pluripotent stem cells (hPSCs) to directly derive hTSCs (Dong et al., 2020; Guo et al., 2021), preimplantation blastoids that contain trophectoderm (bTE) (Yanagida et al., 2021; Yu et al., 2021; Zhao et al., 2021 preprint) and hTSCs derived from preimplantation blastoids (bTSC) (Liu et al., 2021; Yu et al., 2021). All of these emerging models express trophoblast stem cell markers, including TFAP2C, GATA2, GATA3, and TEAD4 (Dong et al., 2020; Guo et al., 2021; Yanagida et al., 2021; Yu et al., 2021); however, the essential transcription factor network required for hTSC self-renewal is currently unknown.

To address these issues, we constructed a continuous pseudotime trajectory of human trophoblast development from zygote to mid-gestation. We computationally predicted the core transcription factors controlling CTB identity *in vivo* and determined their functional requirement for hTSC self-renewal *in vitro*.

## RESULTS AND DISCUSSION

### A molecular atlas of human trophoblast development to mid-gestation

**Fig. 1.**
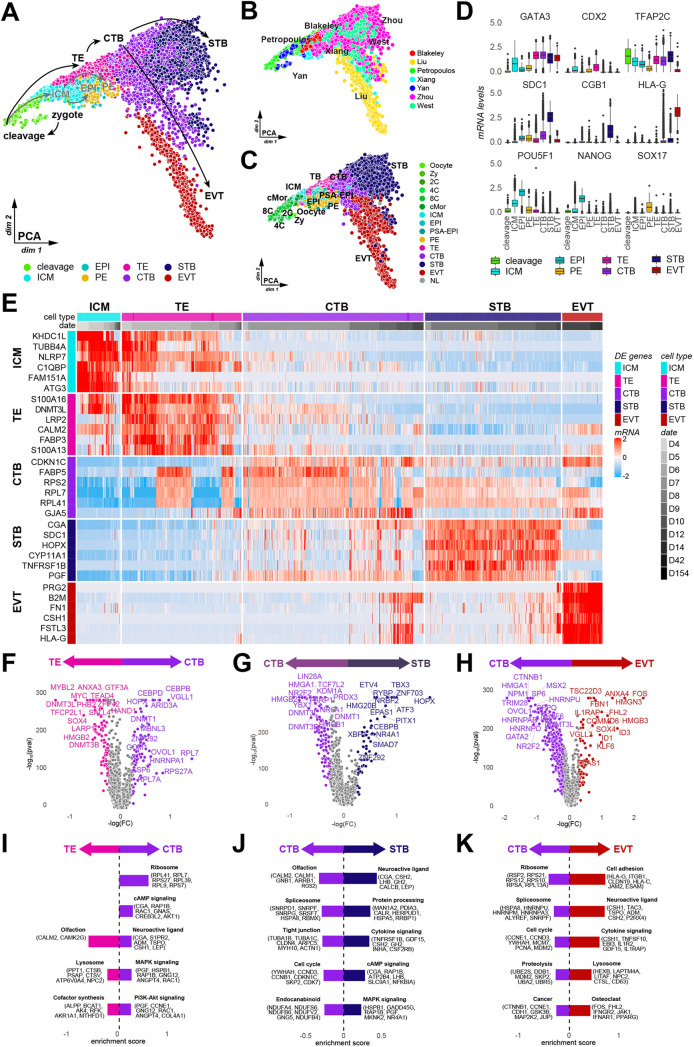
**A molecular atlas of human trophoblast development to first trimester placenta.** (A) PCA for merged single-cell RNA-seq datasets. (B,C) PCA of the combined dataset coloured by original dataset (B) and original labels (C). NL, no label. (D) Normalized read counts for developmentally relevant genes. Box plots show median values (middle bars) and first to third interquartile ranges (boxes); whiskers indicate 1.5× the interquartile ranges; dots indicate outliers. (E) Row normalized read counts of unbiased lineage marker genes. (F-H) Differentially expressed genes from TE versus CTB (F), CTB versus STB (G) and CTB versus EVT (H). (I-K) Enriched KEGG terms for differentially expressed genes between TE and CTB (I), CTB and STB (J) and CTB and EVT (K).

To track trophoblast lineage specification during human embryogenesis, we integrated six Smart-seq2 single-cell transcriptome datasets from zygote to the 24th week of pregnancy (Fig. S1A-D). Specifically, 29 EPI, PE and TE cells from Blakeley et al. (2015), 1529 preimplantation cells from Petropoulos et al. (2016), 952 first and second trimester CTB, STB and EVT from Liu et al. (2018), 548 *in vitro* postimplantation culture ICM, EPI, PE, CTB and EVT from Xiang et al. (2020), 124 cleavage stage, ICM and hESCs from Yan et al. (2013), 139 ICM, TE, CTB and STB from West et al. (2019), Yan et al. (2013) and 5911 *in vitro* postimplantation culture EPI, PE and TE cells from Zhou et al. (2019) (Fig. 1A,B; Fig. S1A). The resulting compendium (Table S1) consisted of 9059 full-length Smart-Seq2 transcriptomes, which separated according to lineage (Fig. 1C) and developmental time (Fig. S1B), and is available as an online resource (http://131.111.33.80:3838/TBatlas/).

We performed shared nearest neighbour clustering on the combined dataset and annotated clusters based on lineage markers (Fig. 1D; Fig. S1C). Preimplantation TE featured pronounced expression of *CDX2*, *TEAD4* and *HAND1* (Fig. S1C) (Hemberger et al., 2020; Knöfler et al., 2002; Niakan and Eggan, 2013). Postimplantation CTB was characterised by *GATA2*, *GATA3*, *OVOL1* and *KRT7* and lacked expression of pluripotency (*POU5F1*, *SOX2*, *NANOG*) and hypoblast (*SOX17*, *HNF1B*) markers (Fig. 1E; Fig. S1C). Differentiated STB exhibited strong enrichment for pregnancy hormones *CGA*, *CGB1* and *LHB*, and EVT specifically expressed *HLA-G*, a mediator of maternal-fetal immune tolerance (Apps et al., 2009; Moffett and Loke, 2006). We observed significant overlap between preimplantation TE and ICM (Fig. 1E; Fig. S1C), supporting the recently reported similarity between preimplantation TE and ICM (Gerri et al., 2020) and plasticity between preimplantation lineages in the human embryo (Guo et al., 2021; Yanagida et al., 2021; Yu et al., 2021). Unsupervised clustering identified *LRP2*, *CALM2* and *FABP3* as preimplantation TE-specific genes and *CDKN1C*, *GJA5* and *NR2F2* as postimplantation CTB markers (Fig. 1E).

To identify potential cell fate regulators, we conducted differential gene expression analysis of transcription factors between trophoblast subtypes (Fig. 1F-H). TE development indicated strong transcriptional similarities between the emerging TE and ICM, including widespread expression of pluripotency factors *TFCP2L1*, *SALL4* and *LIN28A* (Wang et al., 2019; Yang and Moss, 2003) in TE. Naïve pluripotency-associated transcripts were increased in TE compared with CTB, e.g. *TFAP2C*, *ZFP42* and *DNMT3L*, in accordance with trophoblast differentiation from naïve pluripotency (Fig. 1F) (Dong et al., 2020; Guo et al., 2021). Comparison of CTB with STB and EVT identified *NR2F2*, *CTNNB1* and *HMGA1* as candidate regulators for CTB lineage identity, consistent with recent studies (Fig. 1G,H) (Meistermann et al., 2021). To uncover signalling pathways implicated in trophoblast specification *in vivo*, we leveraged stage-specific KEGG pathways analysis (Fig. 1I-K). CTB expressed greater PI3K-AKT and MAPK activators than TE (Fig. 1I), indicating their role in trophoblast maturation. cAMP signalling was enriched in CTB compared with TE, and in STB compared with CTB (Fig. 1I,J), in accordance with cAMP driving STB differentiation (Chang et al., 2005; Wice et al., 1990).

### Pseudotime analysis implicates MAPK signalling in CTB differentiation

**Fig. 2.**
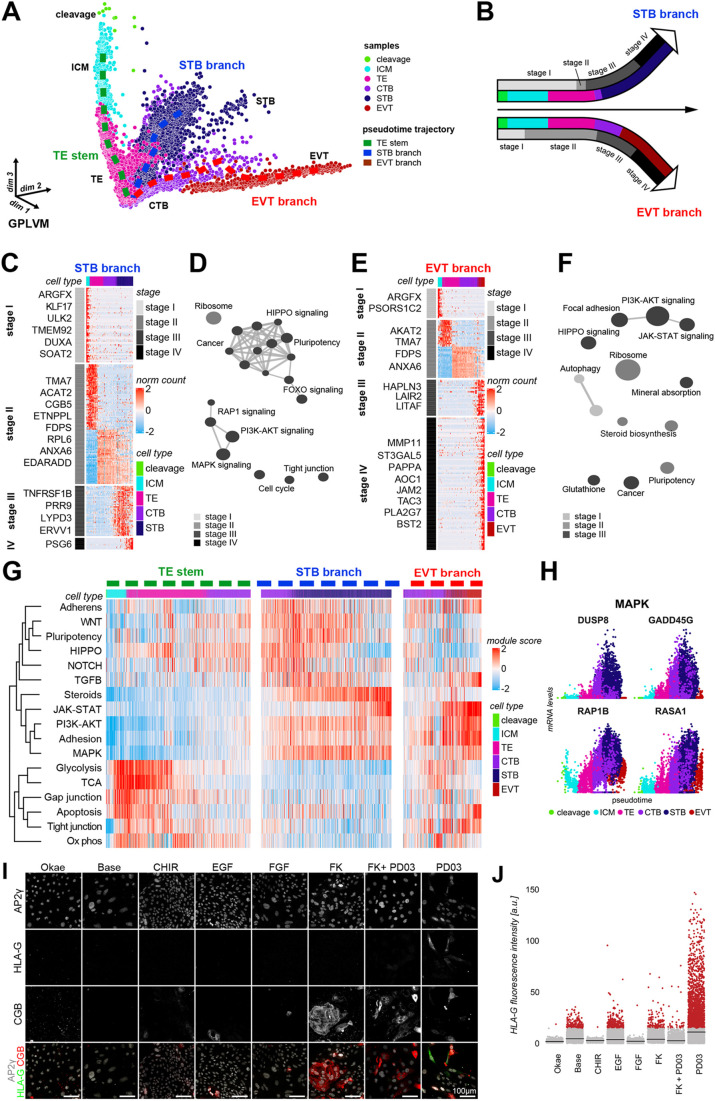
**Pseudotime trajectory implicates MAPK signalling in CTB differentiation.** (A) Pseudotime trajectory (dashed line) within the GPLVM latent space. (B) Schematic of the trophoblast developmental trajectory. (C) Normalized expression counts of the top 95% of genes along the STB branch. (D) Over-represented KEGG terms of the genes in C. (E) Normalized expression counts of the top 95% of genes along the STB branch. (F) Over-represented KEGG terms of the genes in E. (G) Module scores of key pathways in each cell arranged along the pseudotime trajectory. (H) Scaled transcript counts of the most dynamically expressed genes in the MAPK KEGG term. (I) Immunofluorescence of trophoblast (AP2γ), STB (CGB) and EVT (HLA-G) markers in hTSCs cultured with single activators or inhibitors of indicated signalling pathways. Okae, Okae et al. medium; CHIR, CHIR99021; FK, Forskolin; PD03, PD0325901. (J) Quantification of HLA-G fluorescence in indicated conditions (*n*=3).

We employed pseudotime analysis to elucidate the most dynamic phases of trophoblast development with a branch-recombinant Gaussian process latent variable model (GPLVM) (Boukouvalas et al., 2018; Penfold et al., 2017 preprint). The trophectoderm stem (TE stem) consisted of cleavage and blastocyst lineages and divided into STB and EVT branches (Fig. 2A), in agreement with principal component analysis (PCA) (Fig. 1A) and previous reports (Gerri et al., 2020; Meistermann et al., 2021; Vento-Tormo et al., 2018). We derived probabilistic trajectories for both STB and EVT differentiation (Fig. 2B). Importantly, the pseudotimes of individual samples robustly correlated with embryonic age (Fig. S2A), creating a continuous pseudotimeline for human trophoblast development (Fig. 2A,C,E; Fig. S2C,D).

To determine regulatory phases in the STB and EVT trajectories, we identified four key stages associated with the most dynamic changes in gene expression (Fig. 2B). Stage I transcriptomes predominantly expressed genes associated with cleavage for both trajectories (Fig. 2C). In the STB trajectory at stage II, cells undergoing CTB-to-STB transition abruptly increased expression of STB-associated genes such as CGB isoforms and *ACAT2* (Fig. 2C; Fig. S2C) (Hirschmugl et al., 2018; Sasagawa et al., 1987). Among stage III-specific genes, MAPK signalling was significantly enriched (Fig. 2D; Fig. S2B). Genes upregulated at stage IV were candidate factors for STB maturation, including *PSG6* and *PLAC4* (Fig. 2C,D). For the EVT trajectory (Fig. 2E), dynamic genes at stage III included *HAPLN3*, which mediates attachment to hyaluronan, and *LAIR2*, which has been proposed to exhibit an immune modulatory role at the maternal-fetal interface (Apps et al., 2011). Pathway analysis showed an overall increase in focal adhesion-associated transcripts and PI3K-AKT signalling (Fig. 2F; Fig. S2B). To obtain insight into signalling dynamics, we determined module scores for developmentally relevant signalling cascades along the pseudotime trajectories (Fig. 2G). WNT signalling increased from TE to CTB, but gradually decreased during trophoblast differentiation, in agreement with WNT maintaining undifferentiated hTSC cultures (Haider et al., 2018; Okae et al., 2018; Turco et al., 2018). MAPK, TGFβ and PI3K pathway scores increased over pseudotime in both STB and EVT branches (Fig. 2G). EVT enriched for PI3K-AKT signalling activators such as EFNA1 (Fig. S2E). In STB, MAPK enrichment was largely driven by signalling inhibitors, including *DUSP8*, *RAP1B* and *RASA1* (Fig. 2H).

To functionally determine the impact of the most developmentally dynamic signalling pathways on trophoblast lineage acquisition, we conducted an activator/inhibitor screen using hTSCs and quantified differentiation into STB and EVT (Fig. 2I). The cAMP agonist Forskolin (FK) increased STB formation, consistent with previous reports (Okae et al., 2018). Activation of WNT, EGF or FGF/MAPK signalling did not promote either STB or EVT specification, as indicated by the lack of CGB (STB) or HLA-G (EVT) expression (Fig. 2I,J; Fig. S2F-H). Strikingly, mitogen-activated protein kinase kinase (MEK) inhibition via PD0325901 (PD03) induced HLA-G expression within 5 days of induction (Fig. 2I,J; Fig. S2G). PD03-treated cells also showed a trend towards increased CGB expression (Fig. S2F,H). These results demonstrate that MAPK signalling regulates human trophoblast differentiation, which will aid the establishment of efficient protocols for the generation of EVT and STB *in vitro*.

### Okae conditions promote a TE-CTB transition state in hTSCs and blastoids

**Fig. 3.**
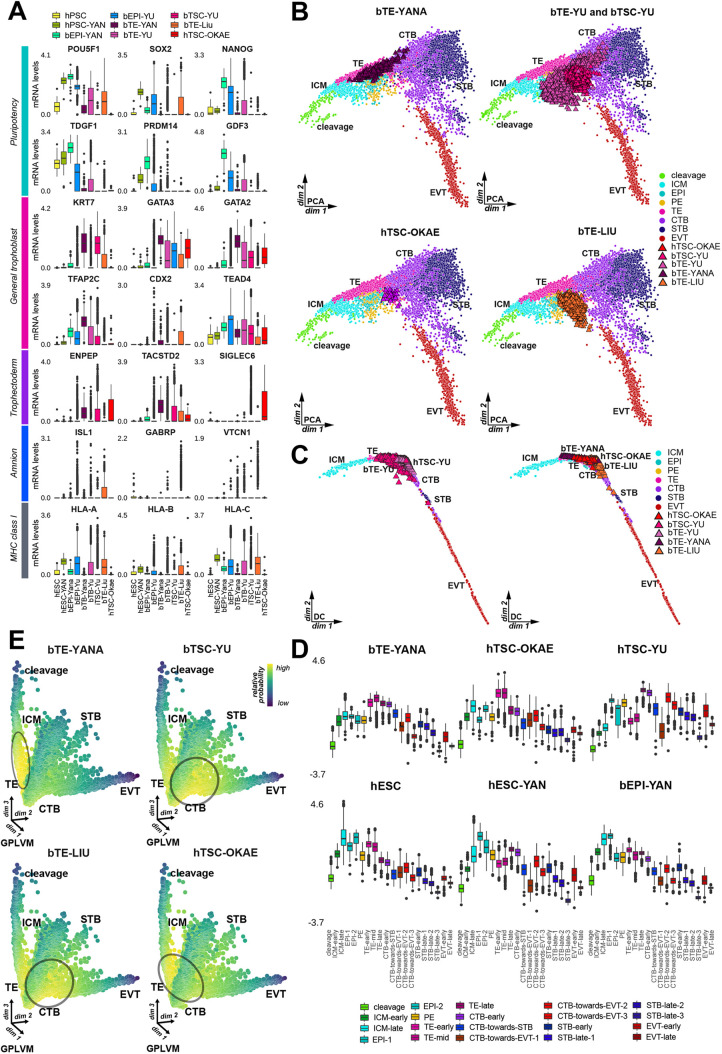
**Okae culture conditions promote TE-CTB transition state in TSCs and blastoids.** (A) Normalized read counts for developmentally relevant genes in *in vitro* cells. (B) PCA projection of *in vitro* cells onto trophoblast developmental trajectory. (C) Diffusion map of *in vitro* cells onto the trophoblast developmental trajectory. (D) Scaled correlation score of *in vitro* cells with trophoblast development lineage subclusters. (E) Relative probability of transcriptomic profile similarity of *in vitro* cells to trophoblast trajectory. Box plots show median values (middle bars) and first to third interquartile ranges (boxes); whiskers indicate 1.5× the interquartile ranges; dots indicate outliers.

hTSCs have been derived from both the blastocyst and first trimester placenta (Okae et al., 2018). Recent studies have demonstrated that naïve PSCs can generate blastoids comprising TE via induction based on Okae conditions (Liu et al., 2021; Yu et al., 2021) or FGF/MAPK and TGFβ inhibition (Kagawa et al., 2022; Yanagida et al., 2021). To determine the developmental stage of trophoblast *in vitro* models and directly compare their developmental progression, we performed single-cell profiling of placenta-derived hTSCs (hTSC-OKAE), hPSCs as a control and re-analysed bTSCs (bTSC-YU) and bTE (bTE-YANA, bTE-LIU and bTE-YU) transcriptomes. Trophoblast cells from all five experimental conditions lacked transcripts for pluripotency regulators and expressed high levels of core trophoblast factors, including *TFAP2C*, *GATA2* and *GATA3* (Fig. 3A). bTE (bTE-YANA, bTE-LIU and bTE-YU) and hTSCs (hTSC-OKAE AND bTSC-YU) exhibited low levels of *HLA-A* and *HLA-B*, and heterogeneous expression of preimplantation trophectoderm-associated genes *CDX2*, *ENPEP*, *TACSTD2*, and *SIGLEC6* (Io et al., 2021) (Fig. 3A).

To quantify the global transcriptional similarity of *in vitro* trophoblast models to placental samples, we integrated bTE-YANA, bTE-YU, hTSC-OKAE, bTSC-YU, bTE-LIU and hPSC transcriptomes into the *in vivo* trophoblast atlas (Fig. 3B,C; Fig. S3A). hPSCs clustered with the pluripotent epiblast (Fig. S3A). hTSC-OKAE cells localised in between TE and CTB (Fig. 3B). bTE-YANA corresponded to an earlier developmental stage and predominantly clustered with preimplantation trophectoderm in both PCA (Fig. 3B) and diffusion maps (Fig. 3C). bTE-YU and bTE-LIU exhibited a slightly later developmental stage (Fig. 3B,C). bTSCs (bTSC-YU) also cultured in OKAE conditions localised to a similar region as hTSC-OKAE cells, but spanned a larger proportion of trophoblast development (Fig. 3B). This result highlights the transcriptional similarity between blastoid and placenta-derived hTSCs. We next clustered each lineage of the *in vivo* trophoblast atlas into substages according to developmental progression (Fig. S3B). This enabled us to calculate correlation scores of *in vitro*-cultured cells towards specific trophoblast subpopulations (Fig. 3D). PSCs correlated best with epiblast populations (Fig. 3D). bTE-YANA exhibited greatest correlation with early-to-mid TE (Fig. 3D). All other *in vitro* lineages displayed high correlation to late TE and early CTB, however bTSC-YU, bTE-LIU and bTE-YU exhibited higher correlation CTB towards EVT and STB subpopulations (Fig. 3B,D; Fig. S3C).

To independently measure developmental time of hTSC and bTEs, we calculated the probability of *in vitro* cells belonging to any given *in vivo* trophoblast transcriptome. We validated this approach by mapping *in vivo* lineages and hPSCs back to the GPLVM framework (Fig. S3D,E). The relative probability of hTSC-OKAEs was highest towards the end of the TE stem, corresponding to the TE-CTB border (Fig. 3E). Equally, bTSC-YU and bTE-YU exhibited broad similarity to the TE-CTB border (Fig. S3E). bTE-YANA showed the greatest similarity to the mid TE stem (Fig. 3E). Gene ontology of bTE-YANA cells showed enrichment for tight junctions, a distinctive feature of TE (Fig. S3F).

We sought to examine the differences between bTE-YANA, hTSC-YU and placenta-derived hTSC-OKAE. Comparative pairwise differential expression analysis showed that bTE-YANA versus hTSC-OKAE exhibited similar changes to TE versus CTB (Table S2). bTE-YANA upregulated preimplantation genes such as *TFCP2L1* and *NLRP7* (Alici-Garipcan et al., 2020; O'Leary et al., 2012) in comparison with hTSC-OKAE (Table S3). Interestingly, gene ontology showed an enrichment of JAK-STAT signalling in hTSC-OKAE, potentially indicating its role in trophoblast maturation (Fig. S3F). These results suggest that the Okae culture regime consistently promotes a late-TE to early-CTB state in both blastoid- and placenta-derived hTSCs.

### The CTB transcription factor network regulates hTSC self-renewal

To identify the transcription factor networks controlling trophoblast lineage identity, we performed weighted gene co-expression network and SCENIC analysis on the integrated trophoblast atlas (Fig. S4A-D; Table S4) (Aibar et al., 2017; Zhang and Horvath, 2005). The CTB module was expressed in both hTSC-OKAE and bTE-YANA, but only bTE-YANA transcriptomes showed enrichment for the preimplantation TE module (Fig. S4B), consistent with their similarity to TE samples in PCA, diffusion maps and probabilistic modelling (Fig. 3B-E).

**Fig. 4.**
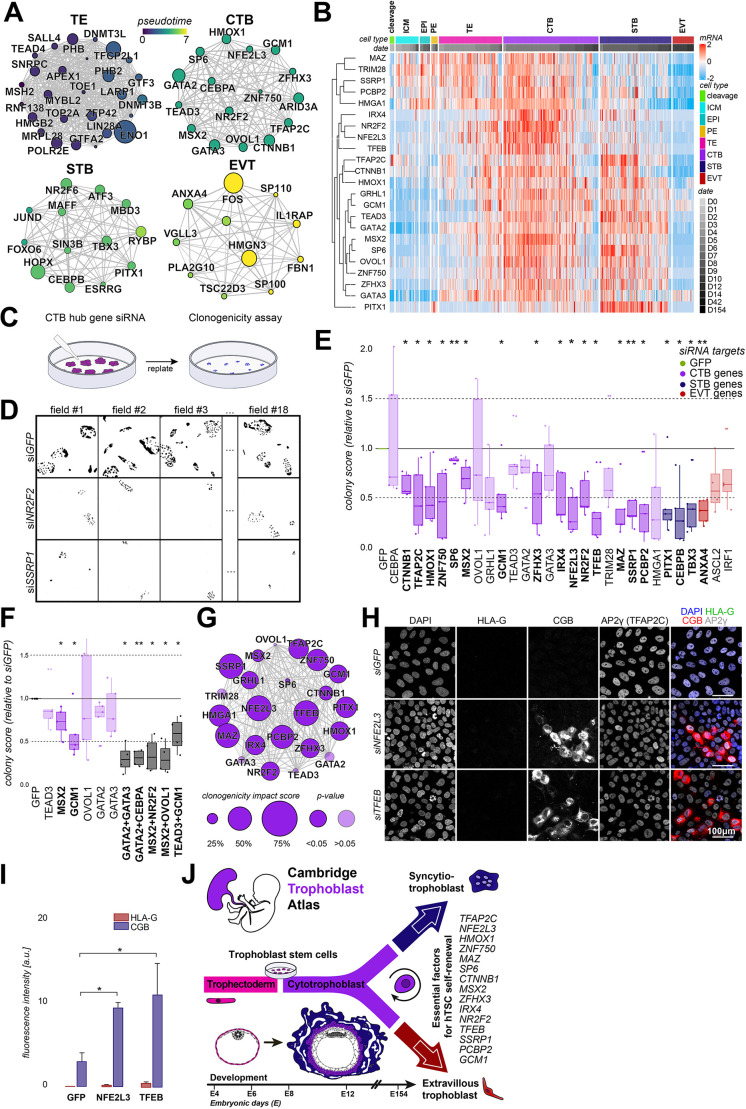
**The CTB transcription factor network regulates hTSC self-renewal.** (A) Transcription factor network associated with each trophoblast cell type. Edge width is proportional to Pearson correlation; node size indicates mean expression; colour shows mean pseudotime of the cell cluster. ICM, gene cluster (GC) 6 and GC9 (Fig. S4A); TE, GC1; CTB, GC4; STB, GC3; EVT, GC8. (B) Heatmap of normalized read counts of transcription factors (TFs) in CTB GC. (C) Schematic of CTB TF siRNA clonogenicity assay. (D) Fluorescent imaging of DAPI in hTSC colonies at day 4 in multiple fields. (E) Normalized number of colonies at day 4 with CTB siRNA. Opacity indicates non-significant changes in clonogenicity (*n*=5). (F) Normalized number of colonies at day 4 with single and dual siRNA treatments (*n*=5). (G) CTB transcription factor network associated with each trophoblast cell type. Edge width is proportional to the Pearson correlation value; node size is proportional to -log(normalized clonogenicity) (*n*=5). (H) Immunofluorescence of siGFP, siNFE2L3 and siTFEB for differentiation markers: STB (CGB) and EVT (HLA-G). (I) Quantification of HLA-G and CGB in GFP and knockout conditions (*n*=3). (J) Graphical summary of results. Significance calculated using a Wilcoxon signed-ranked test (*n*=5). *P<0.01, **P<0.001. Box plots show median values (middle bars) and first to third interquartile ranges (boxes); whiskers indicate 1.5× the interquartile ranges; dots indicate outliers.

We extracted transcription factor networks from the most connected hub genes for TE, CTB, STB and EVT (Fig. 4A). The preimplantation TE network contained *TEAD4*, *LIN28A* and *SALL4*. The CTB network (Fig. 4A,B) centred around key trophoblast lineage markers *GATA2*, *GATA3* and *TFAP2C* and the WNT mediator β-catenin (*CTNNB1*). *SP6*, *MSX2* and *NR2F2* displayed the highest CTB hub scores and we noted a switch from *TEAD4* in TE to *TEAD3* in the CTB network (Fig. 4A,B). NR2F2 is initially expressed in the polar trophoblast and subsequently spreads to all TE by the late blastocyst stage (Meistermann et al., 2021), implicating NR2F2 as an important regulator of CTB initiation. Central hub genes in the STB network included *HOPX*, *CEBPB* and *SIN3B* (Jaremek et al., 2021; Tsuchida et al., 2020), whereas cells differentiating into EVT were characterised by *FOS*, *ANXA4*, *SP100* and hypoxia-inducible *EPAS1* (Knöfler et al., 2019).

To functionally determine the role of the CTB transcription factor circuitry for hTSC self-renewal, we performed a siRNA screen for 24 CTB hub genes followed by an hTSC clonogenicity assay (Fig. 4C). We observed robust knockdown of siRNA targets at the mRNA (Fig. S4E; Table S5) and protein (Fig. S4F-I) level. siRNA transfected hTSCs were replated at low density to examine hTSC self-renewal in the absence of the relevant hub gene (Fig. 4D; Fig. S4J). Knockdown of the majority of CTB hub genes decreased hTSC clonogenicity, with the most detrimental effects observed for *TFAP2C*, *MAZ*, *NFE2L3*, *TFEB*, *PCBP2*, *IRX4*, *GCM1*, *CTNNB1*, *SSRP1*, *NR2F2* and *MSX2* (Fig. 4E). Knockdown of *CTNNB1* and *MSX2* significantly decreased hTSC self-renewal, consistent with our result that CHIR99021 did not promote hTSC differentiation (Fig. 2I) and a recent report showing that *MSX2* represses the STB programme (Hornbachner et al., 2021 preprint). These observations suggest a link between WNT signalling, the core CTB transcriptional network and suppression of STB formation through *MSX2*.

We assessed STB- and EVT-specific transcription factors and found that knockdown of STB hub genes *PITX1*, *CEBPB* and *TBX3* as well as EVT hub gene *ANXA4* also impacted CTB clonogenicity. Surprisingly, the key trophoblast factors *GATA2* and *GATA3* (Castel et al., 2020; Meistermann et al., 2021; Okae et al., 2018) exhibited no significant reduction in hTSC self-renewal. To test whether this could be because of a compensatory mechanism, i.e. a similar set of downstream targets, we performed dual knockdowns of highly correlated CTB transcription factors. Dual knockdown of *GCM1* and *TEAD3* exhibited no greater reduction in clonogenicity than *GCM1* alone (Fig. 4F). In contrast, knockdown of both *GATA2* and *GATA3* reduced clonogenicity by 80.41% compared with GFP-controls (Fig. 4H), suggesting highly overlapping functions for both genes. These data reveal a core transcription factor network for CTB identity (Fig. 4G) and identify 15 essential regulators for hTSC self-renewal *in vitro* (Fig. 4E). To determine whether the observed reduction in clonogenicity was a result of differentiation, we examined the expression of STB (CGB) and EVT (HLA-G) markers in knockdown hTSCs. We discovered that *NFE2L3* and *TFEB* knockdown promoted CGB expression (Fig. 4H,I). Both genes are expressed in late CTB and early STB and decrease in late STB (Fig. S4H), indicating their potential role in regulating early STB differentiation.

Our study elucidates the transcriptional trajectories for human trophoblast development and reveals a pivotal role of MAPK signalling in trophoblast differentiation. We established that both placenta and blastoid-derived hTSCs in OKAE conditions correspond to a developmental stage in between late TE and early CTB, whereas bTE resembles TE. Strikingly, we demonstrated that most hub genes of the CTB transcription factor network are essential for hTSC self-renewal (Fig. 4J). Collectively, our trophoblast compendium provides a rich computational resource to determine the *in vivo* counterpart of *in vitro* cultured cells and an avenue for the systematic interrogation of placental development in our own species.

## MATERIALS AND METHODS

### hTSC culture

Placenta-derived (CT27) hTSCs were propagated in Okae et al. conditions (Okae et al., 2018). Okae medium consisted of 0.3% bovine serum albumin (BSA), 1% insulin-transferrin-selenium-ethanolamine (ITS-X) supplement, 1.5 mg/ml L-ascorbic acid, 50 ng/ml EGF, 2 mM CHIR99021, 0.5 mM A83-01, 1 mM SB431542, 0.8 mM VPA and 5 mM Y27632 in advanced DMEM-F12 basal medium. Cells were cultured on 5 µg/ml collagen IV, in 5% CO_2_ and 5% O_2_. Cells were passaged by dissociation with TryplE (Thermo Fisher Scientific, 12604013) every 3-4 days. hTSCs were tested for contamination, cultured without antibiotic/antimycotic and authenticated for trophoblast markers.

### hPSC culture

All hPSC experiments were approved by the UK Stem Cell Bank Steering Committee and comply with the regulations of the UK Code of Practice for the use of Human Stem Cell Lines. Conventional SHEF6 (International Stem Cell Initiative et al., 2007) were cultured on vitronectin-coated dishes (10 µg/ml; Thermo Fisher Scientific) in Essential 8 (E8) medium (Thermo Fisher Scientific) under hypoxic conditions (37°C, 5% CO_2_, 5% O_2_). Cells were routinely passaged in clumps using 50 mM EDTA. hPSCs were tested for contamination, cultured without antibiotic/antimycotic and authenticated for hPSC markers.

### hTSC differentiation screen

Differentiation media consisted of either Okae conditions or Base medium (0.3% BSA, 1% ITS-X supplement, 1.5 mg/ml L-ascorbic acid) with the respective inhibitor or activator added: 50 ng/ml EGF, 100 ng/ml FGF, 10 mM PD0325901, 0.5 mM A83-01, 2 mM CHIR99021, 2 μM Forskolin or 20 ng/ml Activin. hTSCs were cultured for 5 days in the differentiation media.

### Immunofluorescence

Cells were cultured in μ-Slide 8 wells. Cells were fixed in 4% paraformaldehyde in PBS for 10 min at room temperature and washed three times with PBS. Cells were then permeabilized (0.25% Triton X-100 and 3 mg/ml polyvinyl pyrrolidone in PBS) for 30 min and put in blocking solution (2% donkey serum, 0.1% BSA, 0.01% Tween-20 in PBS) for 15 min. Samples were incubated with primary antibodies in blocking solution overnight at 4°C. AP-2γ (Biotechne, AF5059, 1:250), HLA-G (Bio-Rad, MCA2043, 1:250), hCG (Abcam, ab53087, 1:250), GATA3 (Cell Signaling Technology, 5852, 1:200), KRT7 (Abcam, ab9021, 1:200). Cells were then washed three times in blocking solution and incubated with secondary antibodies [donkey anti-mouse IgG Alexa Fluor 488 (A21202), donkey anti-rabbit IgG Alexa Fluor 555 (A31572), donkey anti-goat IgG Alexa Fluor 647 (A21447), 1:1000, all from Thermo Fisher Scientific] in blocking solution for 2 h. After another three washes in blocking solution, samples were imaged. Donkey serum was acquired from the CSCI, all other reagents were purchased from Thermo Fisher Scientific.

### Image acquisition and analysis

Immunofluorescent images were obtained with either an EVOS M5000 (Thermo Fisher Scientific) or an inverted Leica SP8 confocal microscope. Image analysis was performed using the open-source software Fiji (Schindelin et al., 2012). The significance of changes between experimental conditions was determined using the Kruskal–Wallis test.

### siRNA clonogenicity and differentiation assay

Human siRNAs were designed by and purchased from Horizon Discovery, with four siRNA targeting each gene (Table S6). Then 2 μM siRNA and a 1:5 dilution of Lipofectamine 2000 (Thermo Fisher Scientific) were each incubated in 10 μl Opti MEM (Gibco) for 5 min. siRNA and Lipofectamine solutions were mixed and incubated for 20 min. hTSCs were dissociated for 6 min at 37°C using TryplE. Then 10,000 cells were resuspended in 80 μl Okae medium, mixed with siRNA-Lipofectamine solution and deposited into a 96-well plate. siRNA-Lipofectamine media was replaced with fresh Okae medium 12 h post-plating, and then 60 h post-plating, colonies were either dissociated for 6 min using TryplE and replated in a 12-well dish for clonogenicity or left in the dish for differentiation. Transfected hTSCs were grown for 48 h before fixation in 4% paraformaldehyde in PBS for 10 min at room temperature and washed three times with PBS. Cells were stained with DAPI in PBS for 1 h and washed twice.

### Clonogenicity assay imaging and quantification

The entirety of the well was imaged at 2× power on an EVOS M5000 Imaging System. The IdentifyPrimaryObjects module in CellProfiler 4.0.6 was used to segment the DAPI+ nuclei using the default parameters. Typical diameter of objects was set to be between 60 and 1000 to remove artefacts. Colony number was quantified using DBSCAN clustering, using the Python function sklearn.cluster. Nuclei with an area larger than 400 pixels were discarded. Colonies with nuclei number outside the range of 6 to 300 nuclei were removed.

### Reverse transcription and quantitative polymerase chain reaction

RNA extraction was performed using a Total RNA Miniprep Kit (Monarch). Complementary DNA was obtained with GoScript Reverse Transcriptase (Promega). Quantitative polymerase chain reaction was performed using SYBR green PCR Master Mix (Thermo Fisher Scientific) in a StepOnePlus Real-Time PCR machine (Applied Biosystems). Results were normalized to the geometric mean of UBC and ACTB using the dCt method (Vandesompele et al., 2002).

### Single-cell Smart-seq 2 profiling of hTSCs and hPSCs

Placenta-derived (CT27) Okae et al. hTSCs and SHEF6 hPSCs were transferred into individual tubes containing Smart-seq2 single-cell lysis buffer and immediately frozen in dry ice. RNA separation was performed using biotinylated oligo-dT30VN-tailed oligonucleotides (IDT) conjugated to Dynabeads Streptavidin C1 (65001, Invitrogen) in an RNAse-inhibitor (RNAsin; N2615, Promega) supplemented buffer solution. For transcriptome libraries, complementary DNA (cDNA) was synthesised by reverse transcription using Superscript II (Invitrogen, 200 U/μl) and template-switching oligos (TSO; Exiqon, 100 μM) in 5× Superscript II first strand buffer (Invitrogen) containing RNAse-inhibitor (Promega, 1 U/μl), MgCl_2_ (Invitrogen, 1 M), Betaine (Sigma-Aldrich, 5 M), DTT (Invitrogen, 100 mM) and dNTPs (Roche, 10 mM). Subsequently, material was amplified by PCR using the Kapa HiFi HotStart Readymix (KK2601) and IS PCR primers (IDT, 10 μM). Sample clean-up was performed using AMPure XP beads (A63881, Beckman Coulter) at room temperature, using 80% ethanol, and cDNA samples were eluted in 20 μl elution buffer (Qiagen). For quality control, the DNA concentration of 11 randomly chosen samples per plate was measured using the Agilent Bioanalyser high sensitivity chip system (5067-4626, Agilent Technologies) according to the manufacturer's protocol. Following successful quality control indicated by cDNA between 0.5 and 3 kb, a tagmentation reaction was performed using the Nextera XT DNA kit (FC-131-2001, Illumina). Samples were indexed using the Nextera XT 96-index kit (Illumina) and adapter-ligated fragments were amplified using the Nextera PCR master mix according to the manufacturer's instructions. According to their quality, measured by the Bioanalyser, sample volumes equivalent to a concentration in the range of 200-500 pg were collected and pooled. Following a two-step library purification of the pooled samples with Ampure XP beads and 80% ethanol solution at room temperature (1:0.5 ratio and 1:0.2 ratio of beads to original volume), cDNA was eluted in 22 μl elution buffer and quality control was performed using the Bioanalyser. Pooled libraries were sequenced on an Illumina HiSeq4000 platform with a read length of PE 150 bp. Data are available at ArrayExpress under accession number E-MTAB-10890.

### Trophoblast atlas datasets

Datasets inclusion criteria were: (1) the scRNA-seq was performed on the Smart-seq2 platform; (2) the dataset contains cells within the trophoblast lineage between pre-implantation trophoblast and second trimester trophoblast. Adaptor sequences and low-quality base calls were trimmed using TrimGalore! (Martin, 2011), mapped to the University of California, Santa Cruz human reference genome hg19 using STARaligner v2.5.4 (Dobin et al., 2013). Samples with less than 100,000 mapped reads and or mapping efficiency less than 40% were excluded. Gene counts were quantified using FeatureCounts v1.6.0 (Liao et al., 2014) as counts per million (CPM). Maternal cells were excluded. Downstream analysis included 29 cells from Blakeley et al. (2015), 952 cells from Liu et al. (2018), 1529 cells from Petropoulos et al. (2016), 548 cells from Xiang et al. (2020), 124 cells from Yan et al. (2013), 139 cells from West et al. (2019) and 5911 cells from Zhou et al. (2019). Individual datasets were normalised using the NormalizeData function in the R package Seurat (Butler et al., 2018).

### Dimensionality reduction and lineage annotations

The input dataset for dimensionality reduction consists of the 2000 most variably expressed genes, as determined by the Seurat function FindVariableFeatures. PCA was performed on the combined *in vivo* dataset without hESCs and hTSCs using prcomp from the R package statistics. To visualise the PCA embeddings of the *in vitro* cultured cells, their transcriptomes were projected onto the first two principal components (PCs) generated from the *in vivo* samples. *In vivo* samples were used as a reference point to gauge *in vivo*-*in vitro* similarities in reduced dimensional space. Shared nearest neighbours (SNN) clustering was used on the first ten PCs to cluster the cells in the combined dataset. Each cluster was annotated based on its lineage gene expression profile, its relationship with other clusters and the original dataset labels. Clusters that belong to the same lineage were merged. Diffusion map was performed on the first ten PCs of the combined dataset using DiffusionMap from the R package destiny (Angerer et al., 2016). Cells from the cleavage stage were excluded for this analysis. *In vitro* cells were projected onto the diffusion map using dm_predict.

### Pathway analysis

Differential gene expression analysis was conducted using the wilcoxauc function from the R package presto. Differentially expressed genes were ranked based on the average log fold change. Functional analysis was conducted using the Kyoto Encyclopaedia of Genes and Genomes (KEGG) and Reactome databases via the R package (Yu et al., 2012). This includes gene set enrichment analysis (GSEA) via the gseKEGG function and over-representation analysis (ORA) via compareCluster. The Seurat function AddModuleScore evaluated the expression levels of genes within a particular signalling pathway, metabolic pathway or a cluster of genes (Tirosh et al., 2016).

### Weighted gene co-expression network analysis

Weighted gene co-expression network analysis (Zhang and Horvath, 2005) was used to examine the transcription factor networks using 2765 human transcription factors (Lambert et al., 2018). Hierarchical clustering was performed on this matrix to obtain co-expressed gene networks using default parameters. minModuleSize argument was set to 35 as empirically determined to prevent overclustering. The module scores were used to quantify the expression levels of each network in each cell (Tirosh et al., 2016). In each network, each gene was ranked by its intra-modular connectivity or hub score. The hub genes of each network were defined as the genes with the top five highest hub scores.

### SCENIC analysis

SCENIC version 0.11.0 (Aibar et al., 2017) was used to infer regulon activities in each cell except the *in vitro* samples. The input consisted of 9013 cells. Among the top 10,000 variably expressed genes in this group of cells, the genes for which the mean normalised expression was below 0.05 were removed. The final number of genes used as input was 5337. SCENIC was performed using the Python implementation, on 10 Intel(R) Xeon(R) CPUs with 20 threads. To investigate the relationships between regulons, hierarchical clustering was performed on the regulon activities.

### Pseudotime trajectory and switch point construction

To construct the trophoblast developmental trajectory, a GPLVM (Ahmed et al., 2019; Titsias and Lawrence, 2010) was used to project the 4000 most variably expressed genes across cleavage (compacted morula), ICM, TE, CTB, STB and EVT onto a three-dimensional latent space. Elpigraph in the STREAM package was used to infer the branch assignment of each cell in this latent space (Chen et al., 2019). Final pseudotime trajectories were constructed by branch recombinant GPLVM (B-RGPLVM) (Penfold et al., 2017 preprint) using a Matérn 3/2 covariance function.

The switch points of the 4000 most variably expressed genes were clustered by Gaussian Mixture Models (GMM) using the Mclust function from the R package mclust (Scrucca and Raftery, 2015). To determine the optimal number of clusters, 20 GMMs were built with the cluster number varying from 1 to 20. The final model, with a cluster number of 7, was selected according to Bayesian Information Criteria (BIC).

### Divergence analysis

To analyse the divergence of the STB and EVT branches, the 2-Wasserstein distance between the two corresponding Gaussian Process trajectories (Mallasto and Feragen, 2017) was computed:

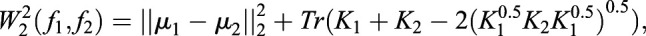
where 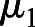 and 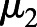 are the means of the Gaussian Processes and *K*_1_ and *K*_2_ are the covariances along the STB and EVT branches, respectively. A diagonal covariance was assumed in this study.

### Cell-cell similarity

Two measures of cell-cell similarity were used: (1) probability of matching the gene expression profile of one cell to a reference; (2) cell-cell pairwise correlation. For the first similarity metric, the probabilistic distribution of gene expression was inferred along each point along the trophoblast trajectory in the three-dimensional latent space established by GPLVM. As both the Gaussian Process and the noise follow a normal distribution, the distribution of expression of genes is also normal:

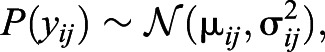
where *y*_*ij*_ is the expression of a gene *i* in a cell *j* along the trophoblast trajectory, *μ*_*ij*_ and σ_*ij*_ are the mean and standard deviation of the inferred expression of a gene *i* in a cell *j*, respectively.

Let *y*_*ik*_ represent the gene expression of another cell *k* that is to be compared with cell *j*. The probability density function of *y*_*ik*_ under the reference distribution is:

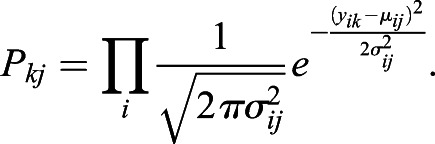
The second measure of cell-cell similarity is the Pearson correlation between *y*_*ij*_ and *y*_*ik*_ over the input genes *i*. The 2000 most variably expressed genes were used to calculate the cell-cell pairwise similarity matrix. Single cell pairwise correlation was calculated between the *in vitro* cultured cells and *in vivo* trophoblast cells. The correlation score was scaled to a mean of 0 and standard deviation of 1 in each *in vitro* cell.

## Supplementary Material

Supplementary information

Reviewer comments
