# Supplementary material for: An integrated atlas of human placental development delineates essential regulators of trophoblast stem cells
Source: Development. 2022 Jul 6;149(13):dev200171. doi: 10.1242/dev.200171 (PMC9340556; doi:10.1242/dev.200171)
Supplement: Supplementary information [file develop-149-200171-s1.pdf]

Figure S1

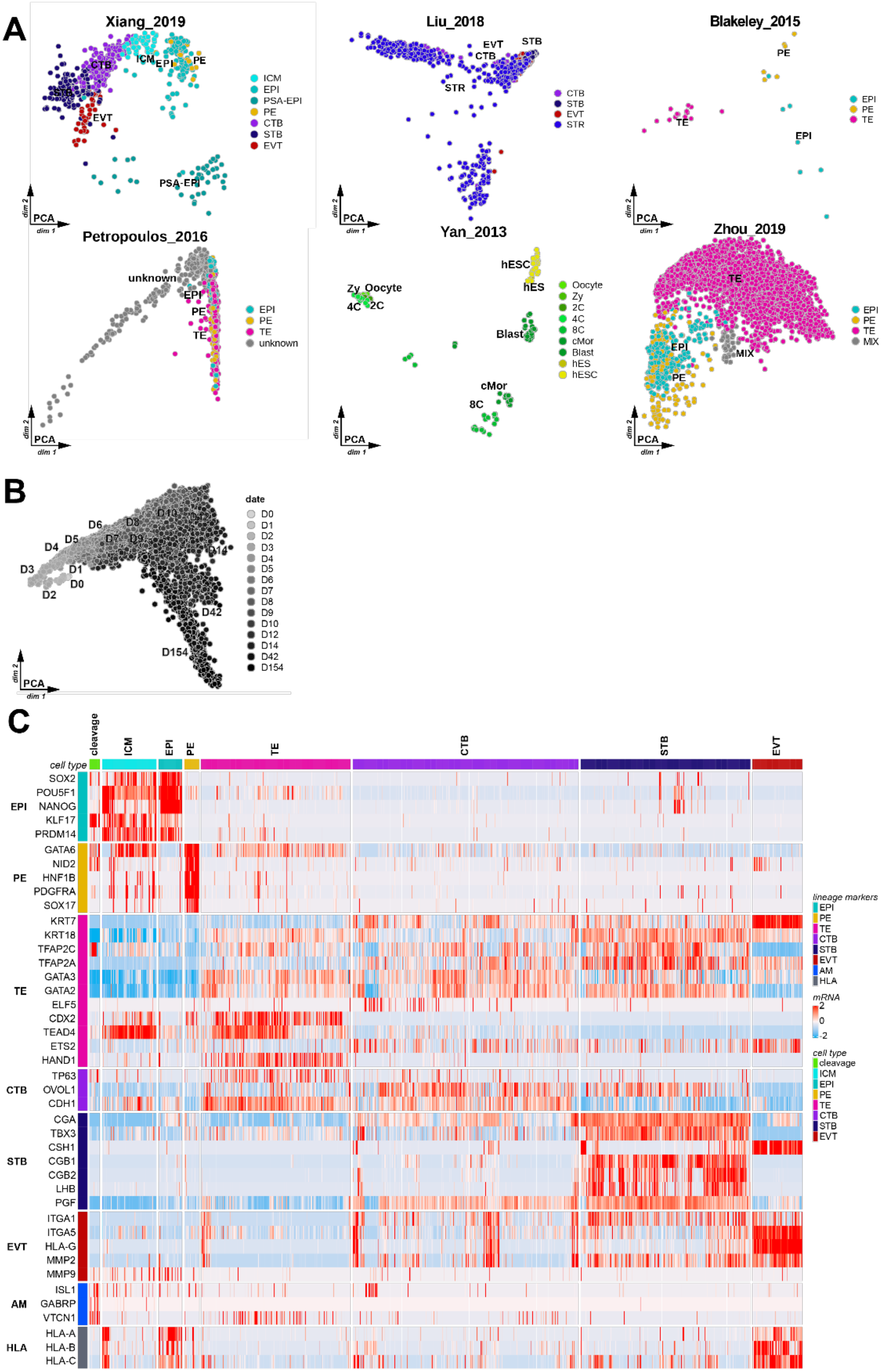

Development • Supplementary information

Fig. S1. Integration of six scRNA-seq datasets into a trophoblast developmental trajectory.

(A) Principal component analysis (PCA) for each of the six datasets used in this study. (B) PCA of the combined dataset coloured by developmental time (D). (C) Normalized expression counts of lineage markers for each annotated lineage.

Development • Supplementary information

Figure S2

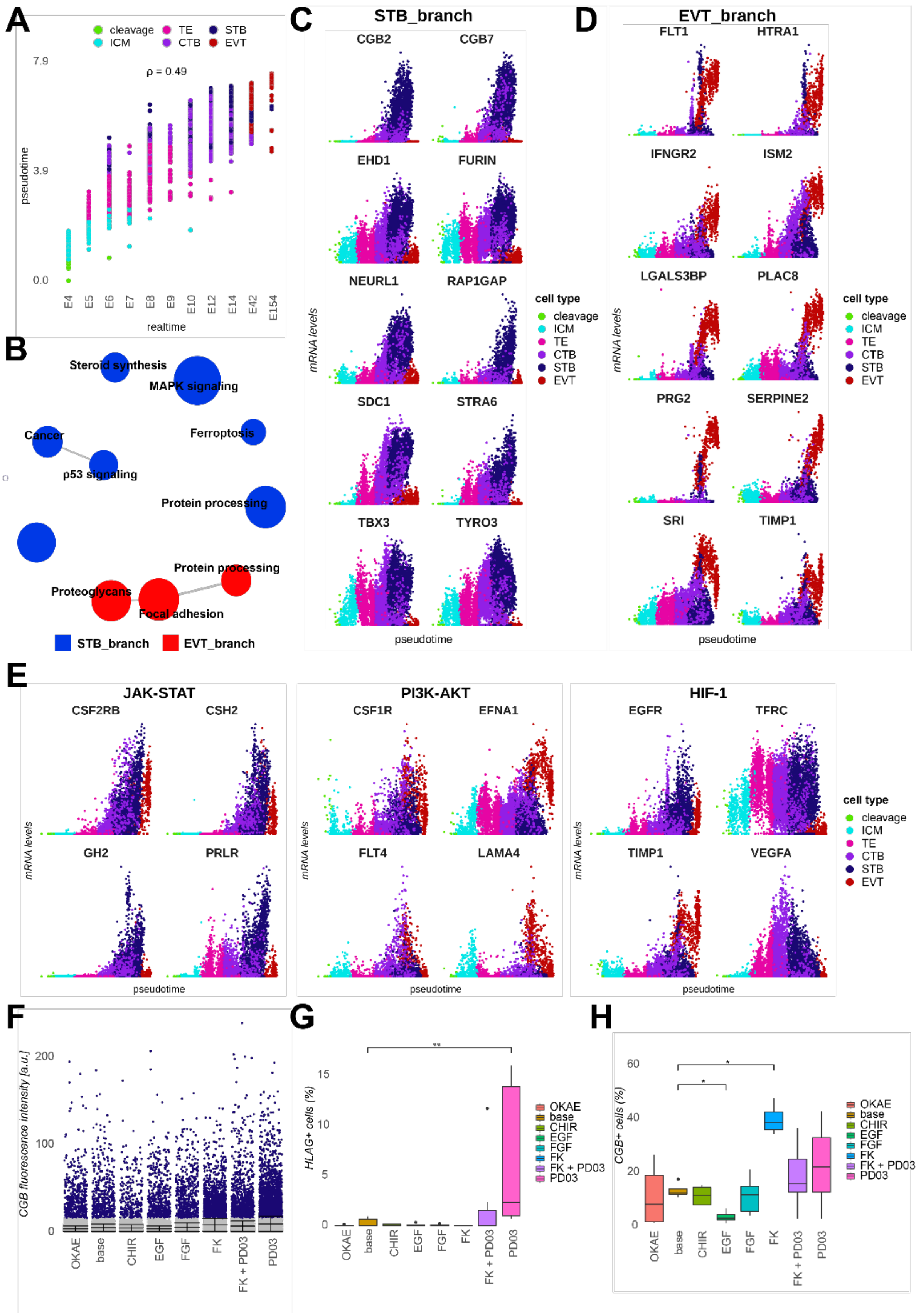

Development • Supplementary information

Fig. S2. Pseudotime trajectory identifies lineage specific signalling pathways. (A) Pearson correlation between pseudotime with embryonic day. (B) Over-represented KEGG terms of significant differentially expressed genes along the STB and EVT branches. ECM: extracellular matrix. (C) Normalized expression counts of the most highly differentially expressed genes along the STB branch over the pseudotime. The counts are scaled to a mean of 0 and standard deviation of 1. (D) Normalized expression counts of the most highly differentially expressed genes along the EVT branch over the pseudotime. Counts are scaled to a mean of 0 and standard deviation of 1. (E) Scaled transcript counts of the most dynamically expressed genes in indicated KEGG terms. (F) Quantification of CGB fluorescence in indicated conditions (n=3). (G) Quantification of HLAG positive cells in indicated conditions (n=3). (H) Quantification of CGB positive cells in indicated conditions (n=3).

Development • Supplementary information

Figure S3

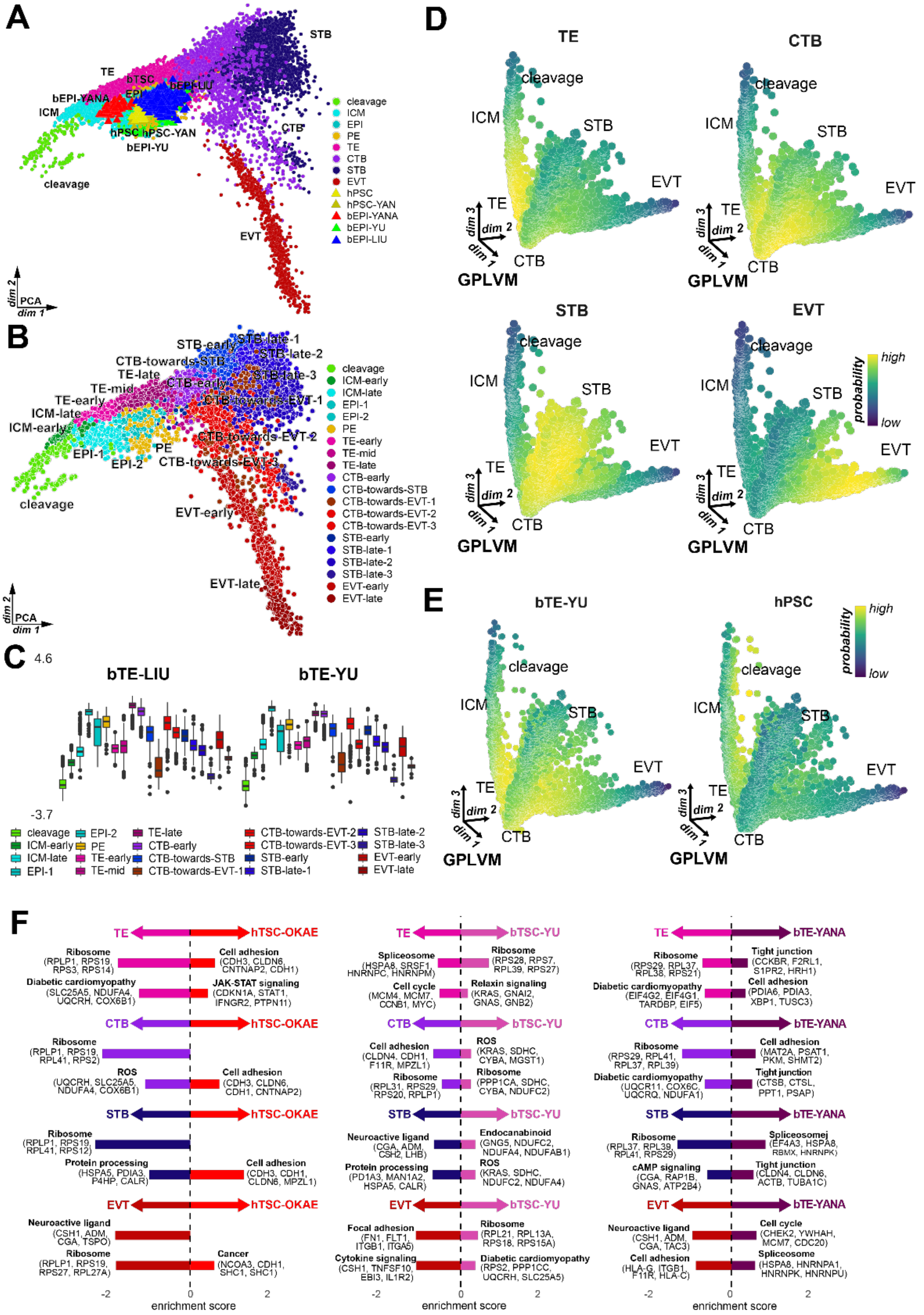
A
STB
TE
bTSC
EPI
bEPI-LIU
bEPI-YANA
ICM
hPSC hPSC-YAN
CTB
bEPI-YU
cleavage
EVT
cleavage
ICM
EPI
PE
TE
CTB
STB
EVT
hPSC
hPSC-YAN
bEPI-YANA
bEPI-YU
bEPI-LIU
dim 2
PCA
dim 1
B
STB-early
STB-late-1
STB-late-2
STB-late-3
CTB-towards-STB
TE-late
CTB-early
TE-mid
TE-early
CTB-towards-EVT-1
ICM-late
ICM-early
CTB-towards-EVT-2
PE
EPI-1
EPI-2
CTB-towards-EVT-3
cleavage
EVT-early
EVT-late
cleavage
ICM-early
ICM-late
EPI-1
EPI-2
PE
TE-early
TE-mid
TE-late
CTB-early
CTB-towards-STB
CTB-towards-EVT-1
CTB-towards-EVT-2
CTB-towards-EVT-3
STB-early
STB-late-1
STB-late-2
STB-late-3
EVT-early
EVT-late
dim 2
PCA
dim 1
C
4.6
bTE-LIU
bTE-YU
-3.7
cleavage
ICM-early
ICM-late
EPI-1
EPI-2
PE
TE-early
TE-mid
TE-late
CTB-early
CTB-towards-STB
CTB-towards-EVT-1
CTB-towards-EVT-2
CTB-towards-EVT-3
STB-early
STB-late-1
STB-late-2
STB-late-3
EVT-early
EVT-late
D
TE
CTB
STB
EVT
cleavage
ICM
STB
EVT
TE
CTB
dim 3
dim 2
dim 1
GPLVM
probability
high
low
E
bTE-YU
hPSC
probability
high
low
cleavage
ICM
STB
EVT
TE
CTB
GPLVM
F
TE
hTSC-OKAE
Ribosome
(RPLP1, RPS19, RPS3, RPS14)
Cell adhesion
(CDH3, CLDN6, CNTNAP2, CDH1)
Diabetic cardiomyopathy
(SLC25A5, NDUFA4, UQCRH, COX6B1)
JAK-STAT signaling
(CDKN1A, STAT1, IFNGR2, PTPN11)
CTB
hTSC-OKAE
Ribosome
(RPLP1, RPS19, RPL41, RPS2)
ROS
(UQCRH, SLC25A5, NDUFA4, COX6B1)
Cell adhesion
(CDH3, CLDN6, CDH1, CNTNAP2)
STB
hTSC-OKAE
Ribosome
(RPLP1, RPS19, RPL41, RPS12)
Protein processing
(HSPA5, PDIA3, P4HB, CALR)
Cell adhesion
(CDH3, CDH1, CLDN6, MPZL1)
EVT
hTSC-OKAE
Neuroactive ligand
(CSH1, ADM, CGA, TSPO)
Ribosome
(RPLP1, RPS19, RPS27, RPL27A)
Cancer
(NCOA3, CDH1, SHC1, SHC1)
-2
0
2
enrichment score
TE
bTSC-YU
Spliceosome
(HSPA8, SRSF1, HNRNPC, HNRNPM)
Ribosome
(RPS28, RPS7, RPL39, RPS27)
Cell cycle
(MCM4, MCM7, CCNB1, MYC)
Relaxin signaling
(KRAS, GNAI2, GNAS, GNB2)
CTB
bTSC-YU
Cell adhesion
(CLDN4, CDH1, F11R, MPZL1)
ROS
(KRAS, SDHC, CYBA, MGST1)
Ribosome
(RPL31, RPS29, RPS20, RPLP1)
Ribosome
(PPP1CA, SDHC, CYBA, NDUFC2)
STB
bTSC-YU
Neuroactive ligand
(CGA, ADM, CSH2, LHB)
Endocanabinoid
(GNG5, NDUFC2, NDUFA4, NDUFAB1)
Protein processing
(PD1A3, MAN1A2, HSPA5, CALR)
ROS
(KRAS, SDHC, NDUFC2, NDUFA4)
EVT
bTSC-YU
Focal adhesion
(FN1, FLT1, ITGB1, ITGA5)
Ribosome
(RPL21, RPL13A, RPS18, RPS15A)
Cytokine signaling
(CSH1, TNFSF10, EBI3, IL1R2)
Diabetic cardiomyopathy
(RPS2, PPP1CC, UQCRH, SLC25A5)
-2
0
2
enrichment score
TE
bTE-YANA
Ribosome
(RPS29, RPL37, RPL38, RPS21)
Tight junction
(CCKBR, F2RL1, S1PR2, HRH1)
Diabetic cardiomyopathy
(EIF4G2, EIF4G1, TARDBP, EIF5)
Cell adhesion
(PDIA6, PDIA3, XBP1, TUSC3)
CTB
bTE-YANA
Ribosome
(RPS29, RPL41, RPL37, RPL39)
Cell adhesion
(MAT2A, PSAT1, PKM, SHMT2)
Diabetic cardiomyopathy
(UQCR11, COX6C, UQCRQ, NDUFA1)
Tight junction
(CTSB, CTSL, PPT1, PSAP)
STB
bTE-YANA
Ribosome
(RPL37, RPL39, RPL41, RPS29)
Spliceosomej
(EIF4A3, HSPA8, RBMX, HNRNPK)
cAMP signaling
(CGA, RAP1B, GNAS, ATP2B4)
Tight junction
(CLDN4, CLDN6, ACTB, TUBA1C)
EVT
bTE-YANA
Neuroactive ligand
(CSH1, ADM, CGA, TAC3)
Cell cycle
(CHEK2, YWHAH, MCM7, CDC20)
Cell adhesion
(HLA-G, ITGB1, F11R, HLA-C)
Spliceosome
(HSPA8, HNRNPA1, HNRNPK, HNRNPU)
-2
0
2
enrichment score

Fig. S3. Staging trophoblast *in vitro* cells using pseudotime

(A) PCA projection of hPSCs onto the trophoblast developmental trajectory. (B) PCA of different lineage subclusters. (C) Scaled correlation score of indicated *in vitro* cells with trophoblast development lineage subclusters. (D) Relative probability of matching the transcriptomic profile of *in vivo* lineages to the trophoblast trajectory. (E) Relative probability of matching the transcriptomic profile of *bTE-YU and hPSCs* to the trophoblast trajectory. (D) Relative probability of matching the transcriptomic profile of bTSC-YU and hPSC to the trophoblast trajectory. (E) Enriched KEGG terms for differentially expressed genes between hTSC-OKAE, bTSC-YU, and bTE-YANA and *in vivo* lineages. (F) Enriched KEGG terms for differentially expressed genes between *in vitro* lineages.

Development • Supplementary information

Figure S4

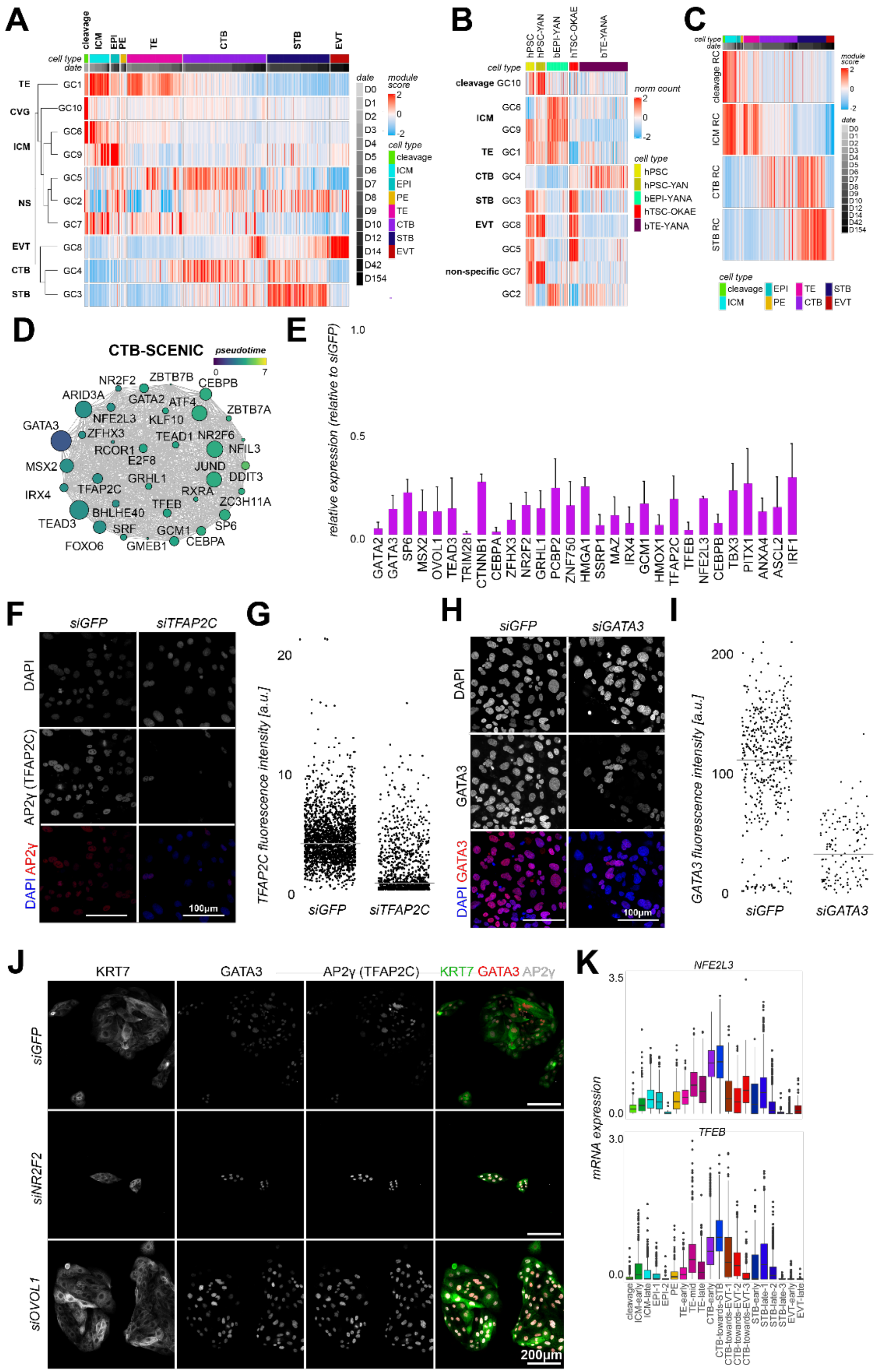

Development • Supplementary information

Fig. S4. Weighted gene co-expression network analysis identified trophoblast cell type specific transcription factor network.

(A) Module scores of transcription factor network in each cell. GC:(transcription factor) gene cluster, CVG: cleavage, NS: non-specific. (B) Module scores of each transcription factor network in each *in vitro* cell. (C) Heatmap of module scores of SCENIC regulon clusters in each cell. RC: transcription factor regulon cluster. (D) SCENIC transcription factor regulon network associated with each trophoblast cell type. Edge width: proportional to the Pearson correlation value; node size: mean gene expression; colour: mean pseudotime. The CTB network is from the CTB RC in Figure S4A. (E) Quantitative PCR of siRNA knockdown of all genes normalized to siGFP on day 3 (n=3). (F) Immunofluorescence of siGFP and siTFAP2C treated hTSCs on day 4. (G) Quantification of TFAP2C fluorescence in indicated conditions (n=3). (H) Immunofluorescence of siGFP and siGATA3 treated hTSCs on day 4. (I) Quantification of GATA3 fluorescence in indicated conditions (n=2). (J) Immunofluorescence of siGFP, siNR2F2, and siOVOL1 for trophoblast lineage markers. (K) mRNA expression of indicated genes from Cambridge trophoblast atlas.

Development • Supplementary information

Table S1. Trophoblast atlas average gene expression
Average gene expression of in vivo and in vitro lineages in trophoblast atlas.

Click here to download Table S1

Table S2. Differential expression analysis between in vivo lineages
Pairwise differential expressed analysis between in vivo lineages.

Click here to download Table S2

Table S3. Differential expression analysis between in vitro lineages
Pairwise differential expressed analysis between in vitro lineages.

Click here to download Table S3

Table S4. Lineage specific transcription factor modules
CTB, STB, and EVT specific transcription factor modules. kWithin: intra-modular connectivity; avgExpr: average expression.

Click here to download Table S4

Table S5. Transcriptional validation of siRNA knockdowns
qPCR results of siRNA KO validation with primer sequences.

Click here to download Table S5

Table S6. siRNA sequences
Target sequences of human siRNA screen.

Click here to download Table S6
