# Supplementary material for: An integrated atlas of human placental development delineates essential regulators of trophoblast stem cells
Source: Development. 2022 Jul 6;149(13):dev200171. doi: 10.1242/dev.200171 (PMC9340556; doi:10.1242/dev.200171)
Supplement: Reviewer comments [file develop-149-200171_review_history.pdf]

An integrated atlas of human placental development delineates essential regulators of trophoblast stem cells

Yutong Chen, Dylan Siriwardena, Christopher Penfold, Adam Pavlinek and Thorsten E. Boroviak
DOI: 10.1242/dev.200171

Editor: Samantha Morris

Review timeline

Original submission:	9 September 2021
Editorial decision:	11 October 2021
First revision received:	17 February 2022
Editorial decision:	21 April 2022
Second revision received:	11 May 2022
Accepted:	12 May 2022

Original submission

First decision letter

MS ID#: DEVELOP/2021/200171

MS TITLE: An integrated atlas of human placental development delineates essential regulators of trophoblast stem cells

AUTHORS: Thorsten Boroviak, Yutong Chen, Dylan Siriwardena, Christopher Andrew Penfold, and Adam Pavlinek

I have now received all the referees' reports on the above manuscript, and have reached a decision. The referees' comments are appended below, or you can access them online: please go to BenchPress and click on the 'Manuscripts with Decisions' queue in the Author Area.

As you will see, the referees express considerable interest in your work, but have some significant criticisms and recommend a substantial revision of your manuscript before we can consider publication. If you are able to revise the manuscript along the lines suggested, which may involve further experiments, I will be happy receive a revised version of the manuscript. Your revised paper will be re-reviewed by one or more of the original referees, and acceptance of your manuscript will depend on your addressing satisfactorily the reviewers' major concerns. Please also note that Development will normally permit only one round of major revision.

We are aware that you may be experiencing disruption to the normal running of your lab that make experimental revisions challenging. If it would be helpful, we encourage you to contact us to discuss your revision in greater detail. Please send us a point-by-point response indicating where you are able to address concerns raised (either experimentally or by changes to the text) and where you will not be able to do so within the normal timeframe of a revision. We will then provide further guidance. Please also note that we are happy to extend revision timeframes as necessary.

Please attend to all of the reviewers' comments and ensure that you clearly highlight all changes made in the revised manuscript. Please avoid using 'Tracked changes' in Word files as these are lost in PDF conversion. I should be grateful if you would also provide a point-by-point response detailing

© 2022. Published by The Company of Biologists under the terms of the Creative Commons Attribution License (https://creativecommons.org/licenses/by/4.0/).

how you have dealt with the points raised by the reviewers in the 'Response to Reviewers' box. If you do not agree with any of their criticisms or suggestions please explain clearly why this is so.

Reviewer 1

Advance summary and potential significance to field

In this article, Chen et al. performed integrative analysis on pre- and post-implantation embryo datasets and placental sample datasets from different gestational ages together with different in vitro trophoblast models, generating a trophoblast cell atlas, which will be available as an online resource. Using this atlas, the authors have revealed the signaling pathways involved in the trophoblast differentiation and identifying key transcription factor networks for hTSC self-renewal, which was validated by clonogenicity following a siRNA knockdown assay. In general, there is no doubt that this can be a great atlas resource for researchers in the field.

Comments for the author

I do have a few questions and additional comments that I hope will help improve the manuscript:
1) The author has integrated six scRNA-seq datasets (inclusion criteria: Smart-seq2 platform) with samples from pre-implantation and in vitro post-implantation stages together with first and second trimester placentas.
Please can you name very clearly in the result section which datasets and which cell types from each datasets were used. I believe that Supp. Figure 1B and C should be in the main figure, as it really helps with the understanding of Figure 1. On this note, it may be advisable to explain why the authors chose to only use smart-seq2 data rather than more datasets, but from different sequencing technologies.
2) Overall, this is a good amount of datasets, but I think there are some additional datasets that should be included to make the atlas more balance and comprehensive. One key dataset that I would suggest to include is the one from West et al PNAS 2019, which specifically examined the CTB, STB and MTB/EVT lineages at the peri-implantation stage. As this study also performed some enrichment for cell types of respective lineages based on some morphological criteria similar to Liu et al Cell Research 2018, and it was also done on Smart-seq2. Another potential dataset is the one from Vento-Tormo et al Nature 2018 which the authors could also extract the cells annotated with the trophoblast lineages for integration into the atlas.

3) For the analysis attempting to integrate trophoblast cells from in vitro models, it was stated: “...We sought to examine the differences between blastoid trophectoderm (bTB-YANA), blastoid-derived TSCs (hTSC-YU) and placenta-derived TSCs (hTSC-OKAE)...” I have several questions on this point:
What are the blastoid trophoblast from Yanagida et al (bTB-YANA), are they the TE in the original paper? If yes, can you clarify this in the manuscript.
For the blastoid-derived trophoblast cells, could the authors explain the reason for not including blastoid TE from Yu et al, but instead only using blastoid trophoblast from Yanagida et al (which as per above, I believe are the TE in the original paper)? From a logical point of view, I think that the blastoid TE from Yu et al should have been included, in addition to the one from Yanagida et al. In summary, why not the TE of Yu et al?, since the TSCs are from Yu et al? and Yu et al, was the first paper describing the generation of human blastoids from Naive ESCs. If there is any technical reason please explain in the text. Are all these added datasets based on smart-seq? My understanding is that bTSC-YU is 10X, but please correct if this is wrong.
Similar to above, why the TE cells from Yu et al 2021 and Liu et al. 2021 were not included? If the reason is due to sequence technology (bulk or 10X), this should be clarified in the text.
Finally, it will be interesting if you could integrate in your analysis the iTSC generated from fibroblasts (Gael et al 2020, Liu et al 2020) as well as TSCs generated from naive ESC to TSC in Cinkornpumin et al 2020. However, if this is not possible for technical reasons such as the data was generated using no-compatible technologies such as 10X or bulk, please comment on it.

© 2022. Published by The Company of Biologists under the terms of the Creative Commons Attribution License (https://creativecommons.org/licenses/by/4.0/).

4) Following with Figure 3 and S3, some of these added datasets have been “projected” rather than integrated. I think that this requires an explanation or even better the same datasets should be integrated using a method that retains the variance of each dataset.
5) The same applies in some way to their primary analysis (Figure 1-2) where batch effect is noticeable. Otherwise, please explain why it was chosen to not use an integration method or a batch removal step.
6) For the siRNA screen of the CTV hub genes, in addition to clonogenicity could the authors also examine the expression of some markers for ST/EVT lineages such as CGB/HLA-G to verify if knockdown of respective hub genes also induces differentiation in the expense of self-renewal capabilities?

Minor points:
In the intro is mentioned that TSCs expressed CDX2 however, as far as this reviewer understand, the Okae media does not maintain CDX2 positive TSCs after initial passages.
Please specify which: commonly used methods for pseudotime analysis do you refer to as not appropriate…it will be interesting to see how one of this perform in your integrated data and compare with the applied method.
Can you please clarify if all the projected cells in Fig3 were smartseq2
For the statement “Preimplantation samples indicated strong transcriptional similarities between the emerging TE and ICM, including widespread expression of pluripotency factors TFCP2L1, SALL4 and LIN28A in TE”. I cannot seem to see the LIN28A annotation in the plot Figure 1D, but instead it was shown in Figure 1E comparing CTB with STB. Can the authors confirm the statement/annotation?
For the statement “We examined STB- and EVT- specific transcription factors and found that knockdown of STB hub genes PITX2, CEBPB and TBX3 as well as EVT hub gene ANXA4 also impacted CTB clonogenicity”, the PITX2 gene is different from what is shown in the figure (PITX1), can the authors please confirm this? Also a figure citation to this statement will be helpful.
Please clarify what do you mean by: "..trophoblast in preimplantation blastoids (Yanagida et al., 2021; Zhao et al., 2021)? Do you mean TE? and blastocyst?
I would highly recommend incorporating a more extensive final paragraph...

Reviewer 2

Advance summary and potential significance to field

The major advance in this report is the integration of datasets from several published studies and the application of cutting edge bioinformatic/computational biological analysis to the data. Insights are gained into potential/candidate regulators and potential/candidate regulatory pathways controlling the trophoblast stem state and the regulation of trophoblast stem cell differentiation and the potential relationships of these cell populations developing in vivo and in a culture dish.

Comments for the author

The merits of this report are in the integration of the published datasets and the bioinformatic analysis. This effort leads naturally to the generation of hypotheses and identification of candidate regulators. The limitations of the work are in the inadequate validation of the findings, the absence of crucial details of experimental design and results, and in some instances sloppy presentation, e.g. the references.
1. At the outset of the Results/Discussion section it was difficult to determine where the sequencing datasets were generated. It was not clear if they were original to the submitted manuscript or whether the sequencing datasets had been previously published. The origin of the datasets became apparent in the Methods section.
2. A major conclusion from the research effort is the importance of specific signaling pathways in trophoblast development. The conclusions are based on a limited experimental survey using a single dose of a pathway inhibitor/activator and immunofluorescence analysis or a measurement of colony size following exposure to an siRNA. The validation of the inhibitor/activator or siRNA is not rigorous enough to generate the conclusions offered by the authors. Reproducibility cannot be readily determined.

© 2022. Published by The Company of Biologists under the terms of the Creative Commons Attribution License (https://creativecommons.org/licenses/by/4.0/).

3. In the report published by Okae and co-workers they described difficulty in transfecting human TS cells. The authors do not provide sufficient descriptions of the methodologies for transfecting siRNAs into human TS cells or the effectiveness of the siRNAs in silencing gene expression. Some data is presented for the siRNA for GATA3; however, the number of replicates performed is not apparent.
4. The references for the manuscript are incomplete.
5. Other comments:
a. the phrase blastoid trophoblast corresponds to trophectoderm is odd - trophoblast cells associated with a blastocyst are trophectoderm by definition
b. Inroduction: Did Haider et al. 2018 and Turco et al. 2018 establish trophoblast stem cell cultures or organoids that likely contained trophoblast stem cells?
c. What is the definition of TB? I would expect it is a generic term for all trophoblast. Consequently, it is confusing to be comparing TB to STB and CTB.
What is a TB-CTB transition state?
d. GPLVM is presented in the Results/Discussion section but not defined until the Methods section.
e. It is probably not a surprise that a common set of culture conditions will select for a specific cellular phenotype.
f. The authors should provide more information about the siRNA targets including sequence information and most importantly their effectiveness in human TS cells.

First revision

<u>Author response to reviewers' comments</u>

We would like to thank the reviewers for their positive assessment of our work and their constructive suggestions. In the course of the revisions, we have performed additional experiments, included new datasets and re-analysed all of the data. This has corroborated our original conclusions and we believe that we were able to address all of the reviewer’s comments.

Reviewer 1
Question 1

- *Name in the result section which datasets and which cell types from each datasets were used.*

We thank the reviewer for their comment and have added the relevant information to the results section.

- *Supp. Figure 1B and C, should be in the main figure, as it really helps with the understanding of Figure 1.*

We thank the reviewer for their suggestion and have integrated Figure S1B,C as Figure 1B,C.

- *Explain why the authors chose to only use smart-seq2 data rather than more datasets, but from different sequencing technologies.*

The reviewer is correct that our in vivo trophoblast compendium is comprised of six datasets sequenced on the Smart-Seq2 platform. Indeed, there are some datasets containing first-second trimester trophoblast transcriptome samples derived from the 10X platform, such as Vento-Tormo et al 2018. We did not include these data because we found significant batch effects between the Smart-Seq2 data and the Vento Tormo et al. 10X datasets, which failed to resolve even after batch correction. To make the reader aware of this, we included a relevant sentence in the results and discussion section.

To illustrate the problems with the Vento Tormo 10x dataset, we generated Reviewer Figure 1. After data normalisation, we merged, without batch correction, the Vento-Tormo dataset with the rest of the Smart-Seq2 datasets. Then, we performed PCA on the combined dataset. Reviewer Figure 1a shows the first trimester CTB from Vento-Tormo, which failed to align with the first

© 2022. Published by The Company of Biologists under the terms of the Creative Commons Attribution License (https://creativecommons.org/licenses/by/4.0/).

trimester CTB from Zhou et al. or Xiang et al.. This indicates significant batch effects between the Vento-Tormo datasets and Smart-Seq2 datasets. We then attempted to correct this batch effect using CCA-based batch correction methods implemented in Seurat (Reviewer Figure 1b). However, this method seems to eliminate the biological variance between cell types as post-implantation CTB from Vento-Tormo overlaps with pre-implantation TE from Petropoulos et al. 2016.

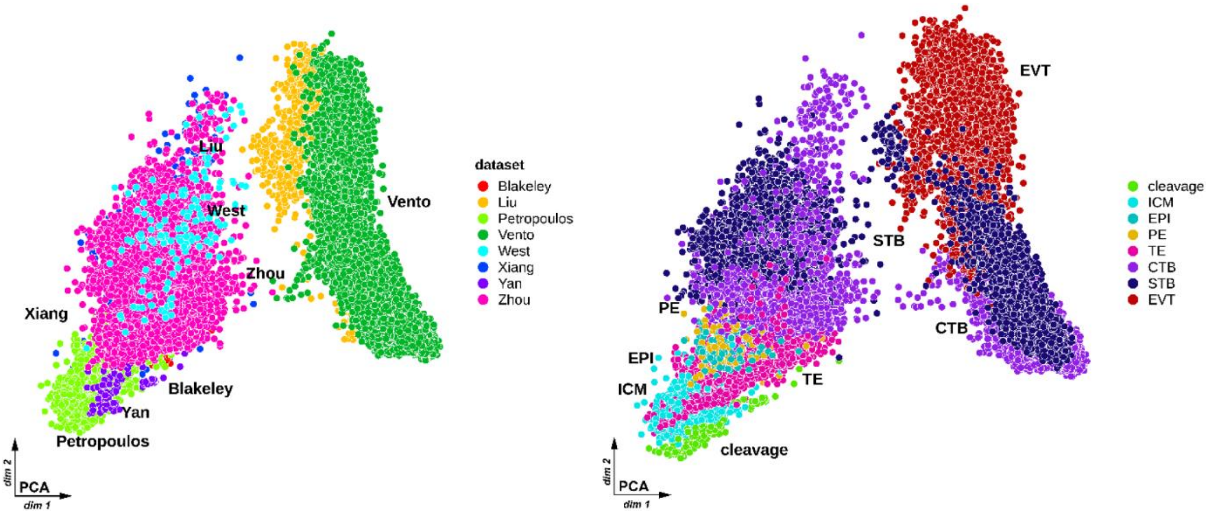

Reviewer Figure 1a: Integration of 10X and smart-seq2 datasets without batch correction

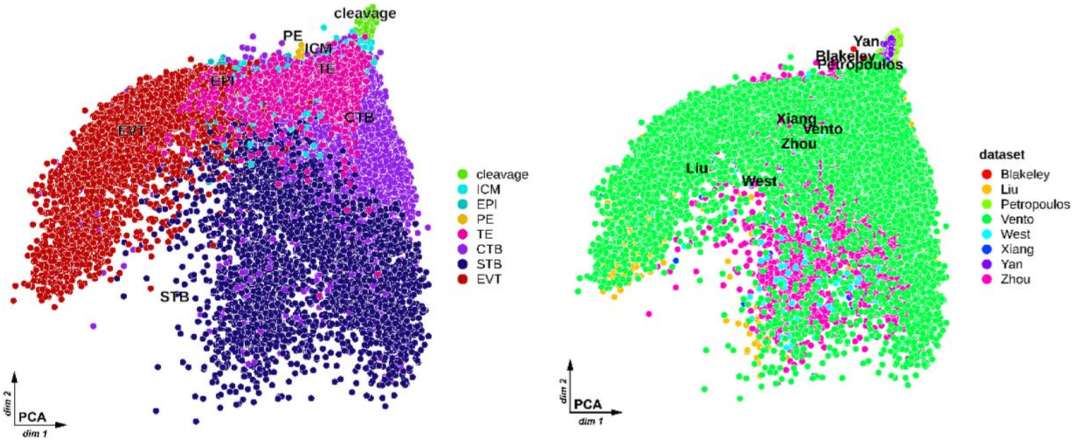

Reviewer Figure 1b: Integration of 10X and smart-seq2 datasets with batch correction

Question 2

- *One key dataset that I would suggest to include is the one from West et al PNAS 2019*

We thank the reviewer for this invaluable suggestion. We have added West et al 2018 into our in vivo trajectory (manuscript Figures 1-3). The results obtained are consistent with our original analysis.

- *Another potential dataset is the one from Vento-Tormo et al Nature 2018*

We agree with the reviewer on the value of Vento-Tormo et al. 2018 dataset. The dataset has two parts: 1) decidual immune cell population derived from the Smart-Seq2 platform, 2) trophoblast cells derived from the 10X platform. We did not consider the first part as the focus of our paper is on the trophoblast lineage. As for the second part, as mentioned in our response to question 1, the batch effects between Vento-Tormo et al. and other Smart-Seq2 datasets have prevented us from integrating this dataset.

Question 3

© 2022. Published by The Company of Biologists under the terms of the Creative Commons Attribution License (https://creativecommons.org/licenses/by/4.0/).

- *What are the blastoid trophoblast from Yanagida et al (bTB-YANA), are they the TE in the original paper? If yes, can you clarify this in the manuscript.*

Yes, we can confirm bTB-YANA in the original manuscript are TE in the original paper. We have clarified our nomenclature in the updated manuscript wherein trophectoderm (TE) is used for preimplantation trophoblast and trophoblast (TB) describes all trophoblast subpopulations.

- *For the blastoid-derived trophoblast cells, could the authors explain the reason for not including blastoid TE from Yu et al, but instead only using blastoid trophoblast from Yanagida et al (which as per above, I believe are the TE in the original paper)?*

We thank the reviewer for pointing out our omission. We have included the blastoid TE from Yu in our analysis (manuscript Figure 3).

Question 4

- *Following with Figure 3 and S3, some of these added datasets have been "projected" rather than integrated. I think that this requires an explanation or even better the same datasets should be integrated using a method that retains the variance of each dataset.*

We thank the reviewer for this valuable comment. In this study, the transcriptomes included can be divided into cells from embryos or placenta (in vivo), and cells derived and cultured from in vitro conditions, such as TSC and ESC. We used the term 'integration' for combining in vivo and in vitro datasets before dimensionality reduction. In contrast, 'projection' is defined as performing dimensionality reduction on the in vivo portion before projecting the in vitro set onto the reduced dimensions. We have clarified this in the methods section.

The rationale of the 'projection' analysis is to use the in vivo samples as a reference point to gauge in vivo-in vitro similarities in reduced dimensional space. Because many dimensional reduction techniques are graph-based, such as PCA, GPLVM and diffuse map, the incorporation of in vitro samples will affect the embedding of in vivo samples in the reduced dimensional space, which distorts the reference point (Reviewer Figures 2a vs 2b). Therefore, to keep the reference point stable, a projection approach is used.

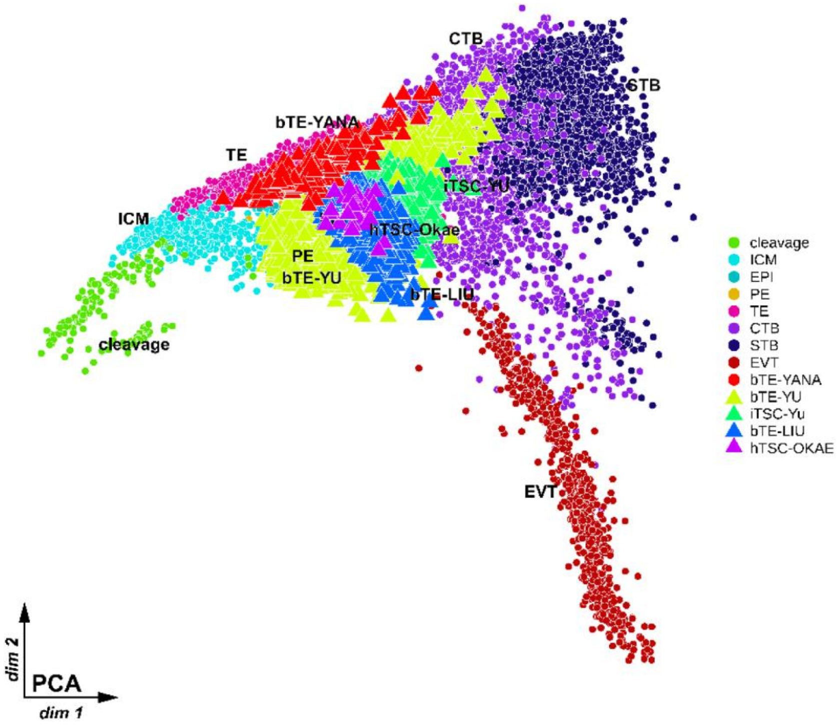

Reviewer Figure 2a: Projection approach. PCA is performed on the in vivo samples and the in vitro samples are projected onto the same PCs.

© 2022. Published by The Company of Biologists under the terms of the Creative Commons Attribution License (https://creativecommons.org/licenses/by/4.0/).

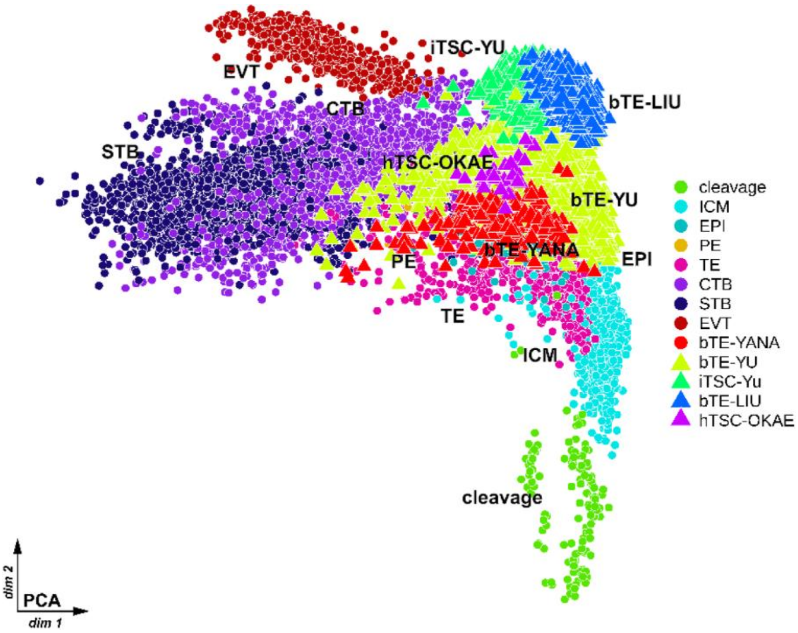

Reviewer Figure 2b: Integration approach. PCA is performed on the combined dataset including in vivo and in vitro cells. Compared with 1a, the incorporation of in vitro samples shifted the topology of in vivo samples.

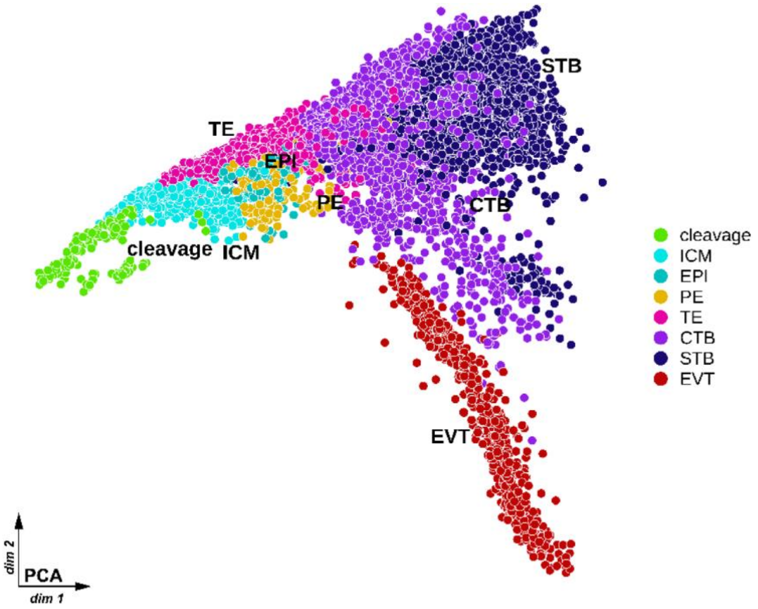

Reviewer Figure 2c: PCA of in vivo cells only.

Question 5

- *The same applies in some way to their primary analysis (Figure 1-2) where batch effect is noticeable. Otherwise, please explain why it was chosen to not use an integration method or a batch removal step.*

We appreciate the reviewer's concern about not performing batch correction. The reason for our choice is that batch correction methods such as shared nearest neighbours require computation of the anchor points. That is, cells that are similar between two datasets. For small datasets such as Blakeley 2015, finding a sufficient number of anchor points is difficult. Thus, these small datasets would not be integrated.

© 2022. Published by The Company of Biologists under the terms of the Creative Commons Attribution License (https://creativecommons.org/licenses/by/4.0/).

Secondly, we noticed minimal batch effect among the smart-seq2 datasets included in this study (Reviewer Figure 2a). This is evident in the close similarity displayed between our freshly sequenced human PSCs and PSCs from Yan 2013 (manuscript Figure 3B).

Third, batch correction can eliminate the biological variance between different datasets. For example, after performing batch correction and PCA (Reviewer Figure 3b), STB and EVT extensively overlap with some parts of the CTB population. This makes it difficult to establish clear developmental trajectories.

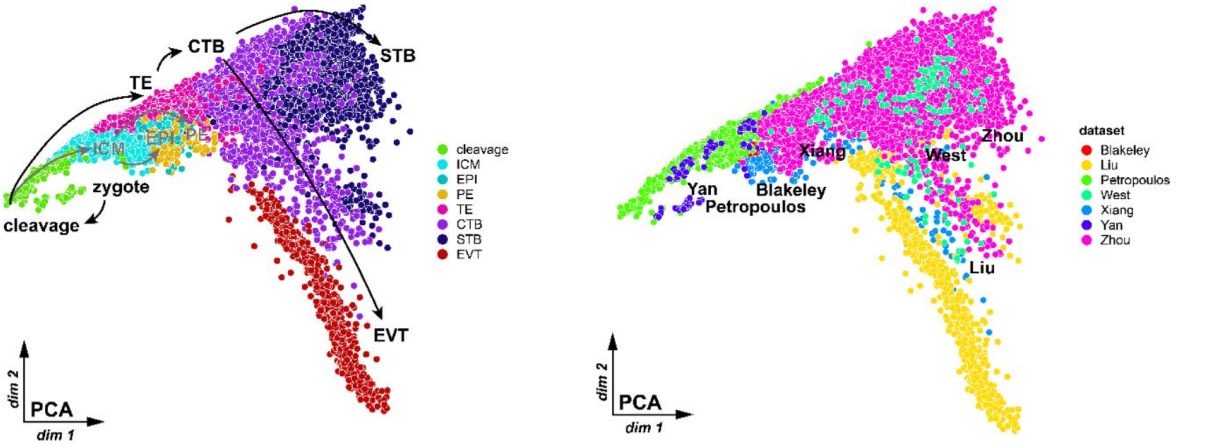

Reviewer Figure 3a: no batch corrections. The results indicate little batch effects, as the same cell types from different datasets are grouped into similar locations in the PCA.

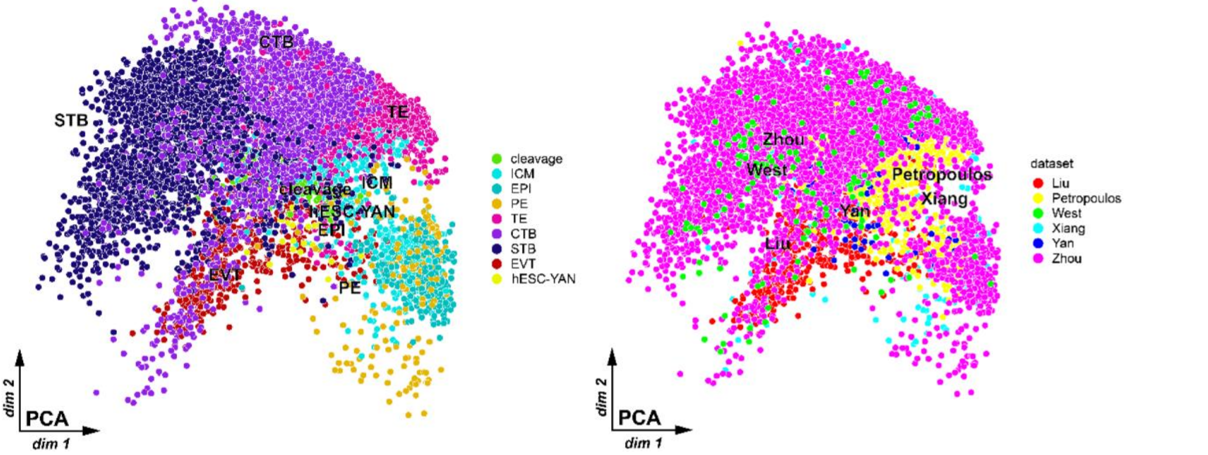

Reviewer Figure 3b: batch corrections using shared nearest neighbours method. The data shows over-correction, as different cell types are grouped into the same location, eliminating the biological variance between, for example, EVT and CTB.

Question 6

- *For the siRNA screen of the CTV hub genes, in addition to clonogenicity, could the authors also examine the expression of some markers for ST/EVT lineages such as CGB/HLA-G to verify if knockdown of respective hub genes also induces differentiation in the expense of self-renewal capabilities?*

We thank the reviewer for their idea. We tested the capability of CTB TF knockdown to induce differentiation in the targets with the lowest clonogenicity. To prevent selection of proliferative TSCs, siRNA treated cells were not passaged at day 2 and allowed to differentiate until day 4 in Okae media. Replacement of Okae media with basal medium proved too stressful to the cells after lipofection, resulting in significant death. Despite the promotion of the stem cell state by the Okae medium, knockdown of NFE2L3 and TFEB demonstrated a significant increase in CGB expression over this timeframe, which can be found in Figure 4H-I in the revised version of the manuscript.

© 2022. Published by The Company of Biologists under the terms of the Creative Commons Attribution License (https://creativecommons.org/licenses/by/4.0/).

Minor points

- *In the intro is mentioned that TSCs expressed CDX2 however, as far as this reviewer understand, the Okae media does not maintain CDX2 positive TSCs after initial passages*

We thank the reviewer for the comment and have updated the manuscript accordingly. Our own studies have shown no expression on a transcriptional level.

- *Please specify which: commonly used methods for pseudotime analysis do you refer to as not appropriate…it will be interesting to see how one of this perform in your integrated data and compare with the applied method.*

We thank the reviewer for the interest in our pseudotime analysis method. Before choosing our approach–BRGPLVM–we tested diffusion pseudotime and the pseudotime method implemented in the package STREAM. Here we illustrate why BRGPLVM is better suited for our dataset. For comparison, we show the results from running B-RGPLVM in Reviewer Figure 4a, and the correspondence between developmental time and pseudotime in Reviewer Figure 5a.

Diffusion pseudotime was not suited on the dataset, because the algorithm failed to assign most of the cells to a particular branch (Reviewer Figure 4b). Furthermore, we noticed a low correspondence between developmental time and pseudotime (Reviewer Figure 5b).

The pseudotime method implemented in STREAM was not suitable either, because the cells were separated into different populations rather than aligning along a continuous axis (Reviewer Figure 4c). We also noticed a low correspondence between developmental time and pseudotime (Reviewer Figure 5c).

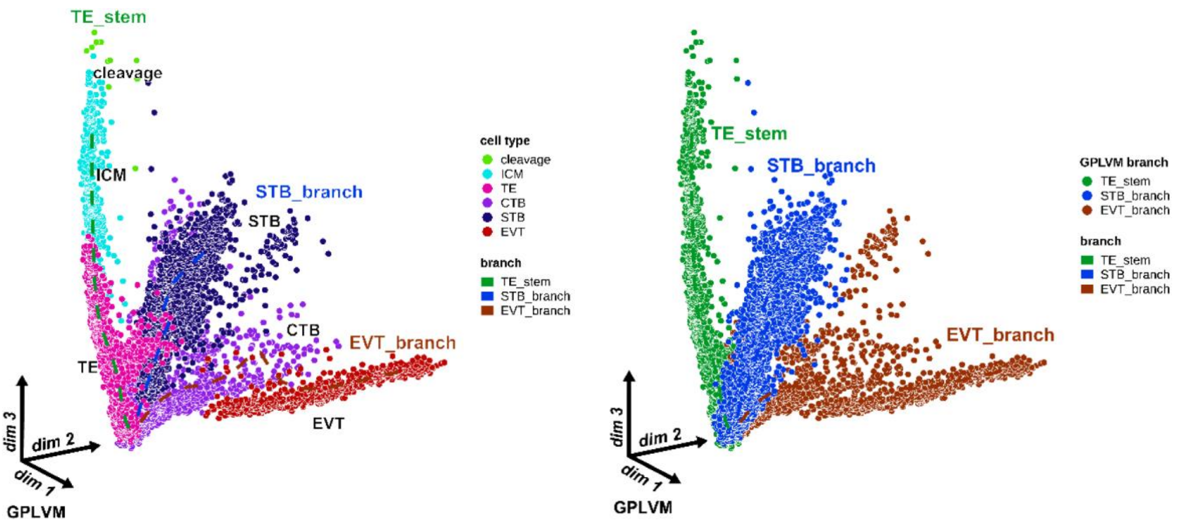

Reviewer Figure 4a: GPLVM

© 2022. Published by The Company of Biologists under the terms of the Creative Commons Attribution License (https://creativecommons.org/licenses/by/4.0/).

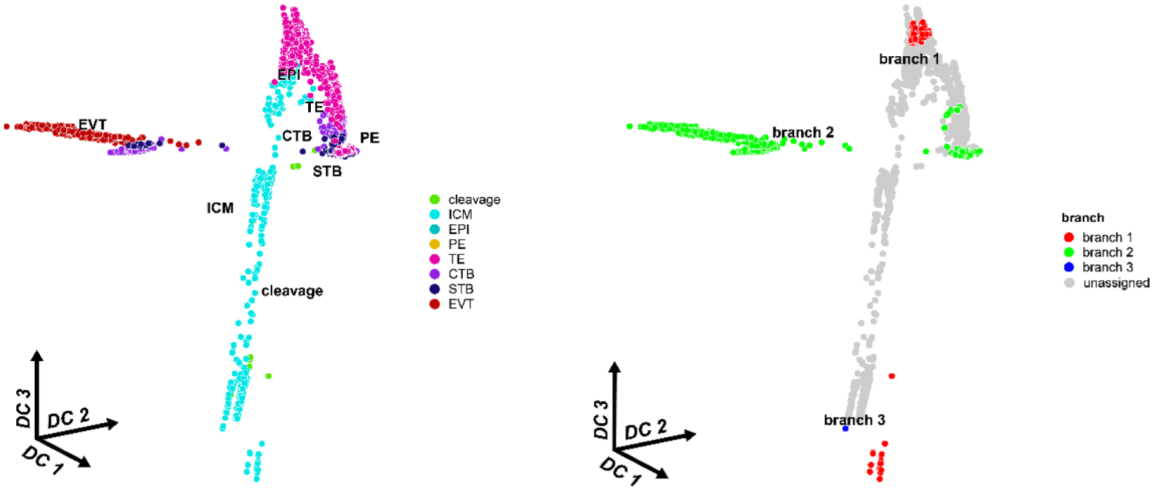

Reviewer Figure 4b: diffusion pseudotime

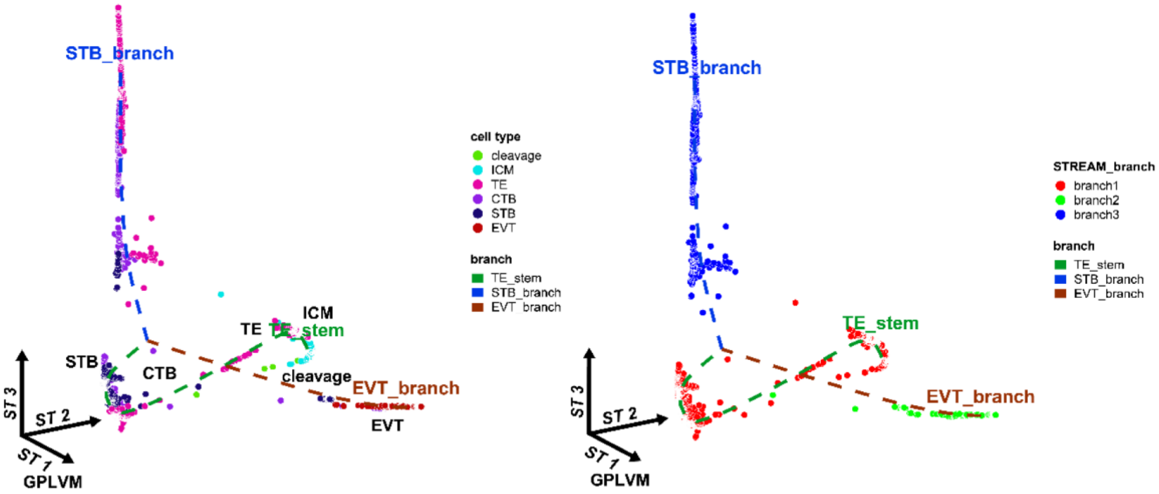

Reviewer Figure 4c: STREAM

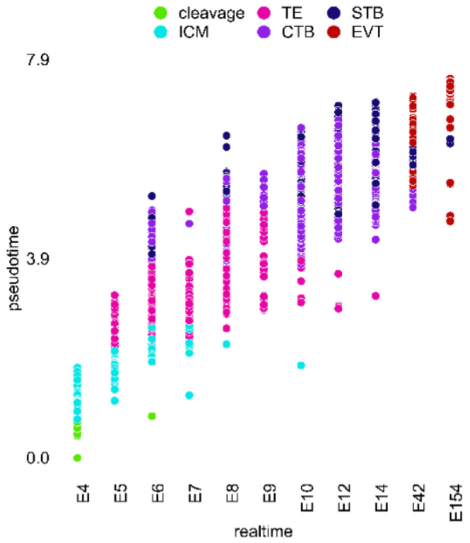

Reviewer Figure 5a: GPLVM

© 2022. Published by The Company of Biologists under the terms of the Creative Commons Attribution License (https://creativecommons.org/licenses/by/4.0/).

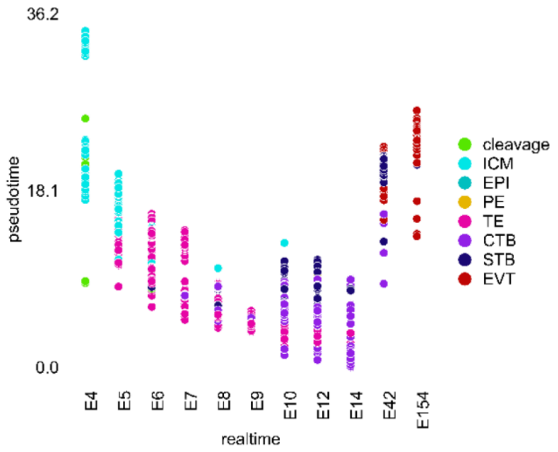

Reviewer Figure 5b: diffusion pseudotime

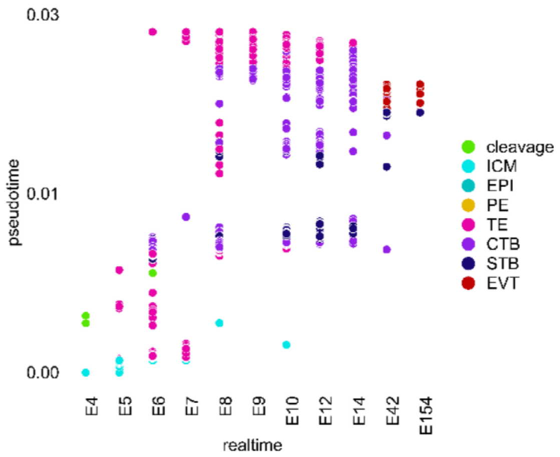

Reviewer Figure 5c: STREAM

- *Can you please clarify if all the projected cells in Fig3 were smartseq2*

Yu (2021) and Liu (2021) are from the 10x platform.

- *For the statement "Preimplantation samples indicated strong transcriptional similarities between the emerging TE and ICM, including widespread expression of pluripotency factors TFCP2L1, SALL4 and LIN28A in TE". I cannot seem to see the LIN28A annotation in the plot Figure 1D, but instead it was shown in Figure 1E comparing CTB with STB. Can the authors confirm the statement/annotation?*

We can confirm that LIN28A was not annotated in Figure 1D. LIN28A expression is present in the TE but is one of the few pluripotency factors that remain in CTB as demonstrated below. This is not the case for all pluripotency factors as shown by TFCP2L1 which is downregulated in CTB (Reviewer Figure 6).

© 2022. Published by The Company of Biologists under the terms of the Creative Commons Attribution License (https://creativecommons.org/licenses/by/4.0/).

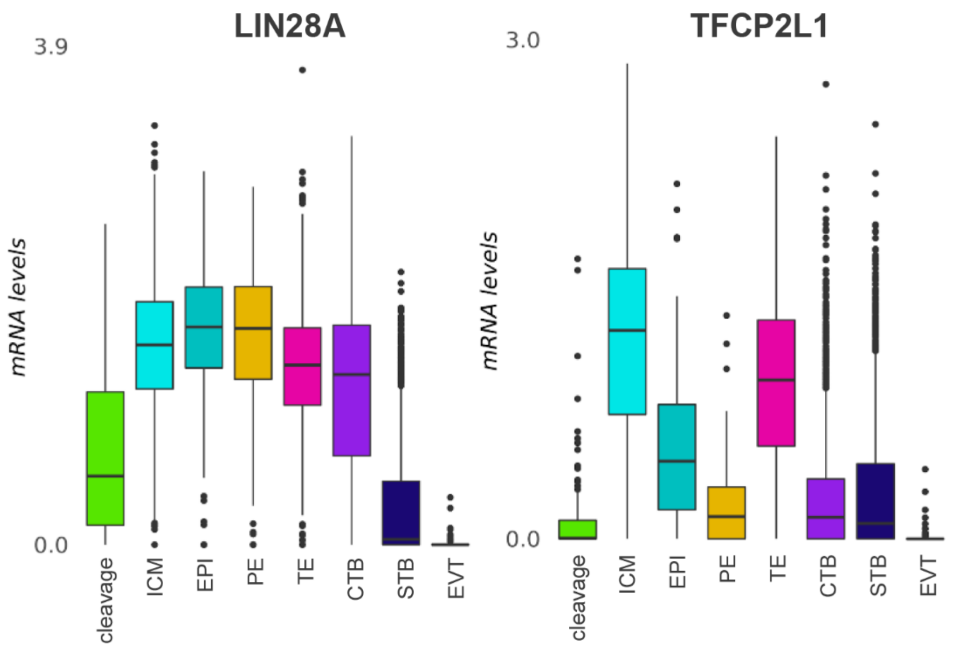

Reviewer Figure 6: mRNA levels of LIN28A and TFCP2L1 across trophoblast trajectory.

- *For the statement "We examined STB- and EVT- specific transcription factors and found that knockdown of STB hub genes PITX2, CEBPB and TBX3 as well as EVT hub gene ANXA4 also impacted CTB clonogenicity", the PITX2 gene is different from what is shown in the figure (PITX1), can the authors please confirm this? Also, a figure citation to this statement will be helpful.*

We thank the reviewer for identifying this error. We confirm PITX1 is the correct gene name.

- *Please clarify what do you mean by: "..trophoblast in preimplantation blastoids (Yanagida et al., 2021; Zhao et al., 2021)? Do you mean TE? and blastocyst?*

Yes, we meant trophectoderm (TE) and have updated the text accordingly.

- *I would highly recommend incorporating a more extensive final paragraph..*

We thank the reviewer for their comment and have integrated a more extensive final paragraph in the revised version of the manuscript.

Reviewer 2

Question 1

- *At the outset of the Results/Discussion section it was difficult to determine where the sequencing datasets were generated. It was not clear if they were original to the submitted manuscript or whether the sequencing datasets had been previously published. The origin of the datasets became apparent in the Methods section.*

We thank the reviewer for their suggestion. In the revised manuscript, we have summarised the included datasets in the first paragraph of the Results/Discussion section to better indicate the origin of each sequencing dataset.

Question 2

- *A major conclusion from the research effort is the importance of specific signaling pathways in trophoblast development. The conclusions are based on a limited experimental survey using a single dose of a pathway inhibitor/activator and*

© 2022. Published by The Company of Biologists under the terms of the Creative Commons Attribution License (https://creativecommons.org/licenses/by/4.0/).

immunofluorescence analysis or a measurement of colony size following exposure to an siRNA. The validation of the inhibitor/activator or siRNA is not rigorous enough to generate the conclusions offered by the authors. Reproducibility cannot be readily determined.

We thank the reviewer for their comment, and we agree that the included validation was incomplete.

The siRNA clonogenicity assay data is based on five independent replicates, which we have clarified in the figure legends of the revised manuscript. For each condition/well, every colony in the well was quantified.

Moreover, we now have included siRNA knockdown validation for each gene (n=3), having achieved a minimum average knockdown of 72% at the transcriptional level (Figure S4E, Table S5 and Reviewer Figure 7).

Sample knockdown efficiency at the protein level for GATA3 and TFAP2C both exceeded 66.4% knockdown efficiency (Figure S4F-I).

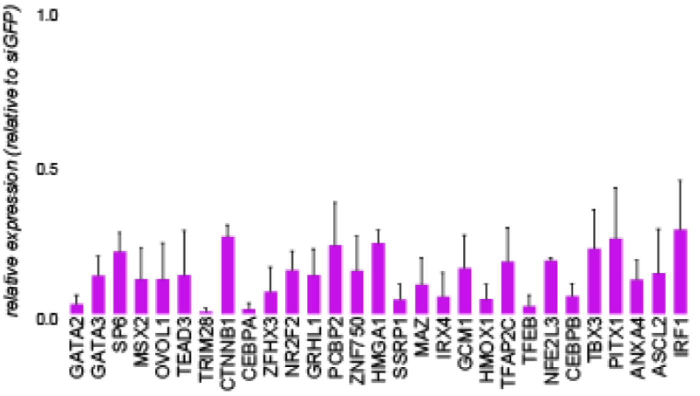

Reviewer Figure 7: Validation of siRNA targets

We further investigated the role of CTB transcription factors on hTSC differentiation by staining knock down hTSCs for STB (CGB) and EVT (HLA-G) markers. Interestingly, we found NFE2L3 and TFEB both significantly increased CGB expression in KO hTSCs (Figure 4H,I).

The inhibitor/activator screen was performed in triplicate, quantifying immunofluorescence intensity across a minimum of 4 fields at 10x magnification.

We would like to respectfully point out that we chose to work with single inhibitors to determine the effects of the relevant pathways on trophoblast differentiation.

The result of increased HLA-G expression upon MAPK inhibition with PD03 was consistently overserved throughout all experiments.

Question 3

- *In the report published by Okae and co-workers they described difficulty in transfecting human TS cells. The authors do not provide sufficient descriptions of the methodologies for transfecting siRNAs into human TS cells or the effectiveness of the siRNAs in silencing gene expression. Some data is presented for the siRNA for GATA3; however, the number of replicates performed is not apparent.*

We thank the reviewer for their comment. We have updated the methods section to give more details on the methodology, included a supplementary table with the siRNA sequences (Table S6), and updated figure captions to indicate replicates. As specified in the original manuscript, we have included a detailed account of our method – including siRNA concentration, lipofectamine

© 2022. Published by The Company of Biologists under the terms of the Creative Commons Attribution License (https://creativecommons.org/licenses/by/4.0/).

concentration, and cell number. As described in our response to Question 2, we have included siRNA validation in triplicate for each gene (Figure S4E-I).

Question 4

- *The references for the manuscript are incomplete.*

We thank the reviewer for their comment and have added further references throughout the manuscript.

Minor points

- *The phrase blastoid trophoblast corresponds to trophectoderm is odd -trophoblast cells associated with a blastocyst are trophectoderm by definition*

We thank the reviewer for their comment and agree the nomenclature in the original manuscript needed clarification. In the updated manuscript bTE describes trophectoderm directly sampled and sequenced from the blastoid (Liu et al., 2021; Yanagida et al., 2021; Yu et al., 2021). This is to differentiate it from bTSC, which describes the sampling and sequencing of hTSCs derived from blastoids (Yu et al., 2021).

- *Introduction: Did Haider et al. 2018 and Turco et al. 2018 establish trophoblast stem cell cultures or organoids that likely contained trophoblast stem cells?*

It is unclear if Haider et al. 2018 and Turco et al. 2018 generated trophoblast stem cell cultures from their organoids, making it more likely the placental organoids contained trophoblast stem cells. We have updated the introduction accordingly.

- *What is the definition of TB? I would expect it is a generic term for all trophoblast. Consequently, it is confusing to be comparing TE to STB and CTB. What is a TB-CTB transition state?*

We thank the reviewer for their comment. In the original manuscript, TE was defined as preimplantation trophoblast. We have updated our nomenclature of preimplantation trophoblast to trophectoderm (TE).

Pseudotime analysis enabled us to place trophoblast cells along a continuous differentiation trajectory from the cleavage stages to STB and EVT (Figure 2A). The TE-CTB transition state refers to the pseudotime that corresponds to the border between the TE and CTB states. The borders between TE and CTB were defined using nearest neighbour clustering. To stage in vitro cells, we determined the relative probability of transcriptomic profile similarity to in vivo cells on the trophoblast trajectory. hTSC-OKAE had the highest similarity with cells on the border between TE and CTB (Figure 3E). This similarity may indicate these cells represent peri-implantation trophoblast.

- *GPLVM is presented in the Results/Discussion section but not defined until the Methods section.*

We kindly wish to point out that we defined GPLVM in the results section:

"We recently developed branch-recombinant Gaussian process latent variable model (GPLVM) (Penfold et al., 2017), which is a probabilistic approach that allows ordering of cells over processes with more than one terminal cell fate."

We have included a more detailed description of how GPLVM was used in Figure 2A in the results section.

© 2022. Published by The Company of Biologists under the terms of the Creative Commons Attribution License (https://creativecommons.org/licenses/by/4.0/).

- *It is probably not a surprise that a common set of culture conditions will select for a specific cellular phenotype.*

We agree with the reviewer that culture conditions promote a specific cellular phenotype. The fact that the culture medium is the master of the cell state, rather than the original source of the cell emphasises the importance of getting the culture conditions right. We hope to illustrate this important concept in our manuscript.

- *The authors should provide more information about the siRNA targets, including sequence information and most importantly their effectiveness in human TS cells.*

We thank the reviewer for their comment. We have included siRNA knockdown validation for all targeted gene (n=3), having achieved a minimum average knockdown of 72% at the transcriptional level (Figure S4E-I, Table S5).

Sample knockdown efficiency at the protein level for GATA3 and TFAP2C both exceeded 66.4% knockdown efficiency (Figure S4E-I).

We have included siRNA sequence information in the updated manuscript (Table S6).

Second decision letter

MS ID#: DEVELOP/2021/200171

MS TITLE: An integrated atlas of human placental development delineates essential regulators of trophoblast stem cells

AUTHORS: Thorsten Boroviak, Yutong Chen, Dylan Siriwardena, Christopher Andrew Penfold, and Adam Pavlinek

I have now received all the referees reports on the above manuscript, and have reached a decision. The referees' comments are appended below, or you can access them online: please go to BenchPress and click on the 'Manuscripts with Decisions' queue in the Author Area.

The overall evaluation is positive and we would like to publish a revised manuscript in Development that meets the Article Length requirement, below.

Reviewer 2

Advance summary and potential significance to field

The authors have expanded our understanding of gene networks controlling development of the human trophoblast cell lineage.

Comments for the author

Thus authors have satisfactorily addressed my concerns.

Second revision

Author response to reviewers' comments

N.A.

© 2022. Published by The Company of Biologists under the terms of the Creative Commons Attribution License (https://creativecommons.org/licenses/by/4.0/).

<u>Third decision letter</u>

MS ID#: DEVELOP/2021/200171

MS TITLE: An integrated atlas of human placental development delineates essential regulators of trophoblast stem cells

AUTHORS: Thorsten Boroviak, Yutong Chen, Dylan Siriwardena, Christopher Andrew Penfold, and Adam Pavlinek
ARTICLE TYPE: Research Report

I am happy to tell you that your manuscript has been accepted for publication in Development, pending our standard ethics checks.

© 2022. Published by The Company of Biologists under the terms of the Creative Commons Attribution License (https://creativecommons.org/licenses/by/4.0/).
